# Innate and adaptive T cells in asthmatic patients: Relationship to severity and disease mechanisms

**DOI:** 10.1016/j.jaci.2015.01.014

**Published:** 2015-08

**Authors:** Timothy S.C. Hinks, Xiaoying Zhou, Karl J. Staples, Borislav D. Dimitrov, Alexander Manta, Tanya Petrossian, Pek Y. Lum, Caroline G. Smith, Jon A. Ward, Peter H. Howarth, Andrew F. Walls, Stephan D. Gadola, Ratko Djukanović

**Affiliations:** a Clinical and Experimental Sciences, University of Southampton Faculty of Medicine, Sir Henry Wellcome Laboratories, Southampton University Hospital, Southampton, United Kingdom; b NIHR Southampton Respiratory Biomedical Research Unit, Southampton University Hospital, Southampton, United Kingdom; c Primary Care and Population Sciences, University of Southampton Faculty of Medicine, Southampton University Hospital, Southampton, United Kingdom; d MantaMatics UG, Geretsried, Germany; e Ayasdi, Palo Alto, Calif; f Novartis Institute of Biomedical Research, Novartis, Basel, Switzerland

**Keywords:** Asthma, T lymphocytes, cytokines, mast cells, phenotype, endotype, regulatory T, T_H_17, T_H_2, mucosal-associated invariant T-cell, ACQ, Asthma Control Questionnaire, BAL, Bronchoalveolar lavage, BNA, Bayesian network analysis, eNO, Exhaled nitric oxide, FOXP3, Forkhead box protein 3, GINA, Global Initiative for Asthma, ICS, Inhaled corticosteroid, IQR, Interquartile range, MAIT, Mucosal-associated invariant T, Tc, Cytotoxic T, TDA, Topological data analysis, Treg, Regulatory T

## Abstract

**Background:**

Asthma is a chronic inflammatory disease involving diverse cells and mediators whose interconnectivity and relationships to asthma severity are unclear.

**Objective:**

We performed a comprehensive assessment of T_H_17 cells, regulatory T cells, mucosal-associated invariant T (MAIT) cells, other T-cell subsets, and granulocyte mediators in asthmatic patients.

**Methods:**

Sixty patients with mild-to-severe asthma and 24 control subjects underwent detailed clinical assessment and provided induced sputum, endobronchial biopsy, bronchoalveolar lavage, and blood samples. Adaptive and invariant T-cell subsets, cytokines, mast cells, and basophil mediators were analyzed.

**Results:**

Significant heterogeneity of T-cell phenotypes was observed, with levels of IL-13–secreting T cells and type 2 cytokines increased at some, but not all, asthma severities. T_H_17 cells and γδ-17 cells, proposed drivers of neutrophilic inflammation, were not strongly associated with asthma, even in severe neutrophilic forms. MAIT cell frequencies were strikingly reduced in both blood and lung tissue in relation to corticosteroid therapy and vitamin D levels, especially in patients with severe asthma in whom bronchoalveolar lavage regulatory T-cell numbers were also reduced. Bayesian network analysis identified complex relationships between pathobiologic and clinical parameters. Topological data analysis identified 6 novel clusters that are associated with diverse underlying disease mechanisms, with increased mast cell mediator levels in patients with severe asthma both in its atopic (type 2 cytokine–high) and nonatopic forms.

**Conclusion:**

The evidence for a role for T_H_17 cells in patients with severe asthma is limited. Severe asthma is associated with a striking deficiency of MAIT cells and high mast cell mediator levels. This study provides proof of concept for disease mechanistic networks in asthmatic patients with clusters that could inform the development of new therapies.

Asthma is characterized by airways inflammation and remodeling. Based on initial studies in animal models^1^ and human T-cell clones^2^ and bronchoscopic studies in patients with mild steroid-naive asthma,^3,4^ it has been viewed as a disease driven by activated T_H_2 cells producing the type 2 interleukins IL-4, IL-5, and IL-13. These cytokines are believed to orchestrate the functions of mast cells,^5,6^ eosinophils,^7^ and IgE-producing B cells/plasma cells. This concept has been challenged with increasing recognition of considerable heterogeneity of asthma mechanisms and definable patient subpopulations associated with immunopathology that cannot be explained by T_H_2 inflammation alone.^8-11^ Discoveries of novel T-cell subsets, notably anti-inflammatory regulatory T (Treg) cells^12,13^ and proinflammatory IL-17–secreting (T_H_17)^14^ and invariant natural killer^15^ T cells, which are implicated in asthma pathogenesis on the basis of studies in animal models and limited evidence in human asthma, have added complexity to our understanding of immunoregulation. Recent studies have revived interest in mast cells,^6,16^ a subject of intense research in the 1980s and early 1990s but not widely seen as candidate targets, possibly because of limited evidence of their role in severe disease.^6,17,18^

Most studies of asthma pathobiology to date have focused on individual or limited numbers of inflammatory cell types, making it difficult to appreciate the cell-cell interactions within complex networks that characterize inflammatory diseases, such as asthma. Therefore we have undertaken a comprehensive assessment of bronchial and circulating T cells, including mucosal-associated invariant T (MAIT)^19^ cells, a novel cell type not yet studied in patients with asthma or other chronic lung diseases, and relevant T_H_2/T_H_1/T_H_17 cytokines, as well as mediators released in the lungs by mast cells and basophils. We have combined these pathobiologic findings with standard clinical parameters used to define asthma severity^20^ and interrogated the rich multidimensional clinicopathobiologic data set using the novel techniques of the machine learning approach (ie, Bayesian network analysis [BNA] and topological data analysis [TDA]).^21,22^ This enabled an in-depth investigation of the roles of individual cell types in relation to asthma severity, characterization of the complex interconnectivity between the diverse clinical and pathobiologic parameters, and identification of clinicopathobiologic clusters that could point to novel asthma endotypes.

## Methods

### Participants

Eighty-four participants (18-70 years) were enrolled from the Wessex Severe Asthma Cohort, NIHR Southampton Respiratory Biomedical Research Unit, and outpatient clinics at University Hospital Southampton: 24 healthy nonatopic participants, 15 patients with mild asthma receiving β_2_-agonists alone, 23 patients with moderate asthma receiving inhaled corticosteroids (ICSs), and 22 patients with severe asthma with persistent symptoms despite high-dose ICSs (n = 16) and oral corticosteroids (n = 6, Table I and see Fig E1 in this article's Online Repository at www.jacionline.org) classified on enrollment according to previously used criteria (see Table E1 in this article's Online Repository at www.jacionline.org).^15^ One hundred seven participants were consented to achieve an approximate minimum group size of 15, according to the physician's assessment of severity at enrollment, which was estimated to provide 80% power at the .05 significance level to detect differences in the primary outcomes of T_H_17 and MAIT cell frequencies in each tissue compartment after loss of missing data.

### Study procedures

Participants were assessed based on history, examination results, skin prick test responses to common aeroallergens, spirometric results, exhaled nitric oxide (eNO) levels, serum IgE levels, and (except for patients with severe asthma) methacholine responsiveness. Lung samples were obtained by means of sputum induction,^23^ bronchoalveolar lavage (BAL), and endobronchial biopsy.^15,17^ By using flow cytometry, the following T-cell subsets were characterized by their surface markers and intracellular cytokines^15^ in the circulation (blood) and lungs (sputum and BAL cell pellets; cells from collagenase-dispersed endobronchial biopsy specimens were used to provide bronchial mucosal cells): T_H_17-cells, T_H_1-cells, and IL-13–secreting-T_H_2 cells; CD3^+^CD4^+^ (or CD8^−^) forkhead box protein 3 (FOXP3)^+^ Treg cells; cytotoxic T (Tc) cells (T_C_1 and T_C_2); γδ-17 T cells; and MAIT cells. Like other authors,^24^ we could not detect with confidence IL-4– or IL-5–secreting T cells using intracellular cytokine staining. Levels of type 1 (IFN-γ, IL-2, and 12p70) and type 2 (IL-4, IL-5, and IL-13) cytokines, IL-10, IL-17A, mast cell proteases, and basophil-derived basogranulin were measured in serum, sputum, and BAL fluid by using standard methods (for details, see the Methods section, Table E2 and Fig E2 in this article's Online Repository at www.jacionline.org).

The study was approved by the Southampton and South West Hampshire Research Ethics Committee B. All participants provided informed consent.

### Statistical analysis

Data were first analyzed by using standard statistical methods, classifying subjects as healthy or having mild, moderate, or severe asthma as defined above and in the Methods section in this article's Online Repository.^15^ BNA was then applied to all the pathobiologic and clinical features to seek association in relation to asthma severity. Finally, TDA was applied to the same data to create a network of distinct clinicopathobiologic clusters.

### Data elaboration and standard statistics

Data distribution was tested by using the Shapiro-Wilk test, and data were logarithmically transformed if they were not normally distributed. For all analyses, 2-tailed *P* values of less than .05 were considered significant. Data were compared between the healthy and control groups (Mann-Whitney *U* or Student *t* tests) and between each asthma severity group and the control subjects (Kruskal-Wallis test or ANOVA), depending on the distribution of the data. For the latter, an overall 5% significance level was adjusted for multiple comparisons by using the Bonferroni method. Groups ranked according to disease severity were tested for linear trend by using polynomial contrasts (or the Jonckheere-Terpstra test, if not normally distributed). Data are expressed as medians with interquartile ranges (IQRs) unless stated otherwise. Correlations were tested by using the Spearman *r* coefficient. Kolmogorov-Smirnov tests identified significant differences between distributions within a single cluster. Data were analyzed with Prism 6.0 (GraphPad Software, San Diego, Calif) and SPSS 21.0 (IBM, Armonk, NY) software.

### Network analyses

For network analyses (BNA and TDA), data were used from 62 participants with the most complete data. Missing data were imputed by using average values specific to each tissue and disease severity subgroup. A composite value was generated for each parameter by using a weighted average across each compartment: sputum and BAL fluid for concentrations of soluble mediators and blood, sputum, BAL fluid, and biopsy specimens for cell counts, providing airway and tissue composite readouts, respectively, with a matrix of 62 participants and 26 pathobiologic and 26 clinical parameters (see the Methods section and Tables E2 and E3 in this article's Online Repository at www.jacionline.org for definitions of terms). Interconnectivity between clinical and pathobiologic parameters was first explored with BNA (Genie 2.0; Decision Systems Laboratory, Pittsburgh, Pa). Data were discretized to describe nonlinear correlations into 2 bins for binary variables or 5 to 9 bins for continuous variables.

### TDA

To use the full range of available clinical and pathobiologic data simultaneously to identify multidimensional features within the data set, which might not be apparent with traditional methods, we used the novel technique of TDA, which is particularly suited to complex biological data sets. This approach represents a high dimensional data set as a structured 3-dimensional network in which each “node” comprises subjects similar to each other in multiple dimensions. Lines or “edges” are drawn between nodes that contain shared data points. Statistical tests can then be performed on groups or features that emerge from the inherent structure of the data set. This method combines features of standard clustering methodologies and also provides a geometric representation of the data.^21,22^ In contrast to most other techniques that depend on prior hypotheses and that focus on pairwise relationships within the data,^25^ this geometric visualization allows recognition of multidimensional features (patterns) within the data in a less supervised, data-driven manner to identify meaningful subgroups that become apparent (self-define themselves) on visualization (please see the TDA plots). In addition, TDA does not require an *a priori* definition of the number of clusters anticipated.

TDA was performed, as previously described,^21^ with IRIS 2.0 software (Ayasdi, Palo Alto, Calif), constructing networks with parameters from Table E3. Three inputs were used: a distance metric, 1 or more filter functions, and 2 resolution parameters (“resolution” and “percent overlap” or “gain”). A network of nodes with edges between them was created by using a force-directed algorithm. The nodes represent bins or “microclusters” of data points, and 2 nodes are connected if their corresponding collections of data points have a point in common.^21^ Variance-normalized Euclidean distance was used as a distance metric, with 2 filter functions: principal and secondary metric singular value decomposition (for further explanation, see the Methods section in this article's Online Repository). Resolution and gain settings were selected where the network structure permits identification of subgroups. Kolmogorov-Smirnov tests identified parameters that differentiate each subgroup from the rest of the structure and create clusters. Comparisons between multiple clusters used 1-way ANOVA, with *post hoc* tests with the Bonferroni correction.

For additional methods used, see the Methods section in this article's Online Repository.

## Results

**Fig 1:**
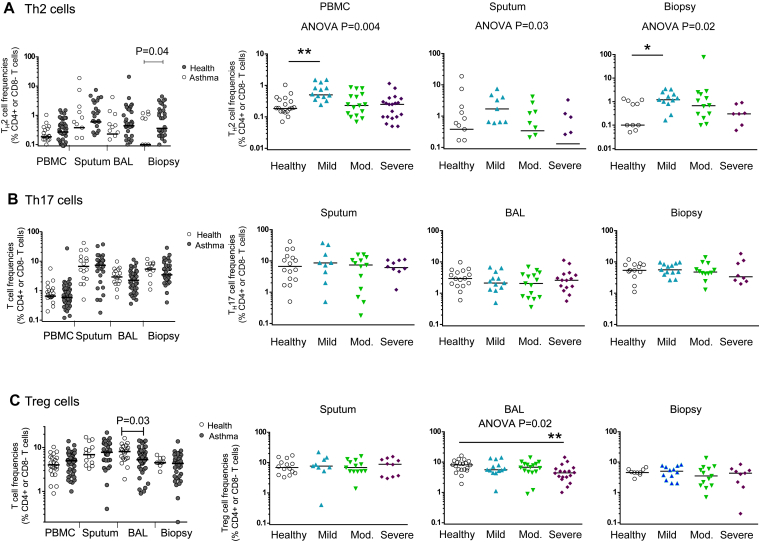
Frequencies of CD3^+^CD4^+^ T cells expressing IL-13 (T_H_2 cells; **A**), IL-17 (T_H_17 cells; **B**), and FOXP3 (Treg cells; **C**) in PBMCs, sputum, BAL fluid, and bronchial biopsy specimens as a percentage of live CD3^+^CD4^+^ T cells or, for endobronchial biopsy specimens, a percentage of CD3^+^CD8^−^ T cells. *Horizontal lines* show medians. *Left columns*, Healthy control subjects versus asthmatic patients with Mann-Whitney *U P* values. *Right 3 columns*, Stratified by disease severity with Kruskal-Wallis *P* values (*P* < .05). *P < .05 and **P < .01, *post hoc* Dunn test compared with healthy subjects.

Data were first analyzed by using standard statistical methods without imputation or composite averages, classifying subjects as healthy or as having mild, moderate, or severe asthma (see Table E1 and the Results section in this article's Online Repository at www.jacionline.org). Previous observations that mild steroid-naive asthma is characterized by a bias toward type 2 inflammation were confirmed, with increased numbers of IL-13–secreting CD4^+^ (T_H_2) cells in sputum, BAL fluid, and endobronchial biopsy specimens from patients with mild asthma (Fig 1, *A*) and ratios of IL-13– to IFN-γ–secreting CD4^+^ (T_H_1) cells (see Fig E3 in this article's Online Repository at www.jacionline.org). However, this bias was not seen in patients with severe asthma, in whom frequencies of IL-13–secreting T_H_2 cells were not significantly different from those in healthy subjects, although we did not measure frequencies of IL-4– or IL-5–secreting T cells. Similarly, in patients with mild asthma, there were significant increases in median concentrations of the type 2 cytokines IL-5 (median, 0.13 pg/mL; IQR, 0.05-0.19 pg/mL) compared with those in healthy subjects (0.003 pg/mL; IQR, 0.001-0.006 pg/mL; *P* < .001) and IL-13 (0.009 pg/mL; IQR, 0.0009-0.026 pg/mL) compared with those in healthy subjects (0 pg/mL [IQR, 0-0.0008 pg/mL]; *P* < .05) in BAL fluid (see Fig E4 in this article's Online Repository at www.jacionline.org). In sputum neither IL-5 nor IL-13 levels were increased in patients with mild or moderate asthma. In patients with severe asthma, IL-5 levels were also significantly increased in both BAL fluid (0.015 pg/mL; IQR, 0.007-0.19 pg/mL; *P* < .05) and sputum (6.18 pg/mL; IQR, 3.13-14.8 pg/mL) compared with levels seen in healthy subjects (1.19 pg/mL; IQR, 1.0-2.2 pg/mL; *P* = .005; see Fig E4, *B*), suggesting that its secretion might be relatively steroid insensitive and might be derived from cellular sources other than airway T_H_2 cells.

Analysis showed no significant differences in T_H_17 cell or γδ-17 T-cell frequencies between asthmatic patients and healthy subjects (Fig 1, *B*, and see Fig E5 in this article's Online Repository at www.jacionline.org). Furthermore, we found no evidence of dysregulation of the T_H_17 response during cold-induced exacerbations of asthma (see the Results section and Figs E6 and E7 in this article's Online Repository at www.jacionline.org). Likewise, IL-17 levels were not increased in BAL fluid, sputum, or serum from asthmatic patients (see Fig E4, *B*, and data not shown; *P* > .05 in all compartments). However, when asthma was stratified according to severity, IL-17 levels in BAL fluid were increased in patients with mild asthma (*P* = .04; see Fig E4, *A*). Increased IL-17 levels were associated with the presence of allergic rhinitis (0.017 pg/mL [IQR, 0.0065-0.043 pg/mL] if present and 0.005 pg/mL [IQR, 0.0033-0.010 pg/mL] if absent, *P* = .02), with airway eosinophilia (*r*_*s*_ = 0.34, *P* = .04) and high serum IgE levels (*r*_*s*_ = 0.42, *P* = .007; see Fig E4, *C*).

T_H_17 cells share functionally and developmentally antagonistic relationships with the immunoregulatory Treg cells that regulate airway hyperresponsiveness in murine asthma models.^26^ We observed a slight but significant deficiency of BAL CD4^+^FOXP3^+^ Treg cells in asthmatic patients (median frequency, 5.3%; IQR, 4.3% to 8.2%) compared with those in healthy subjects (8.1%; IQR, 5.6% to 10%; *P* = .03; Fig 1, *C*), which was restricted to patients with severe asthma (Fig 1, *C*) and was not evident in blood, sputum, or bronchial biopsy specimens.

**Fig 2:**
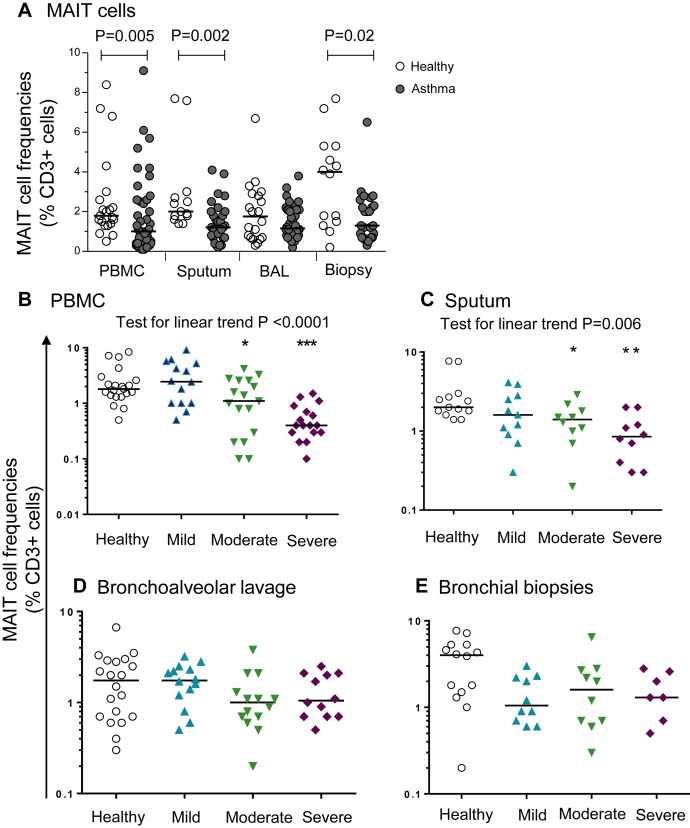
MAIT cells (Vα7.2^+^CD161^+^) as proportions of CD3^+^ T cells in blood, sputum, BAL fluid, and endobronchial biopsy specimens in healthy subjects and asthmatic patients **(A)** and stratified by disease severity **(B)**. *Horizontal lines* show medians. Unpaired *t* tests were used for log-transformed data. MAIT cell deficiency correlates with severity by linear trends across groups using residuals on log-transformed data (where *P* < .05). *P < .05, **P < .01, and ***P < .001, *post hoc* Dunnett test compared with healthy subjects.

There was a striking deficiency of T-cell receptor Vα7.2^+^CD161^+^ MAIT cells in blood, sputum, and biopsy specimens from asthmatic patients, which was related to disease severity and treatment with ICSs (Fig 2 and see Fig E8 in this article's Online Repository at www.jacionline.org). There was evidence of seasonal variation in MAIT cell frequencies and association with serum vitamin D3 concentrations and use of oral corticosteroids (see the Results section and Fig E9 in this article's Online Repository).

**Fig 3:**
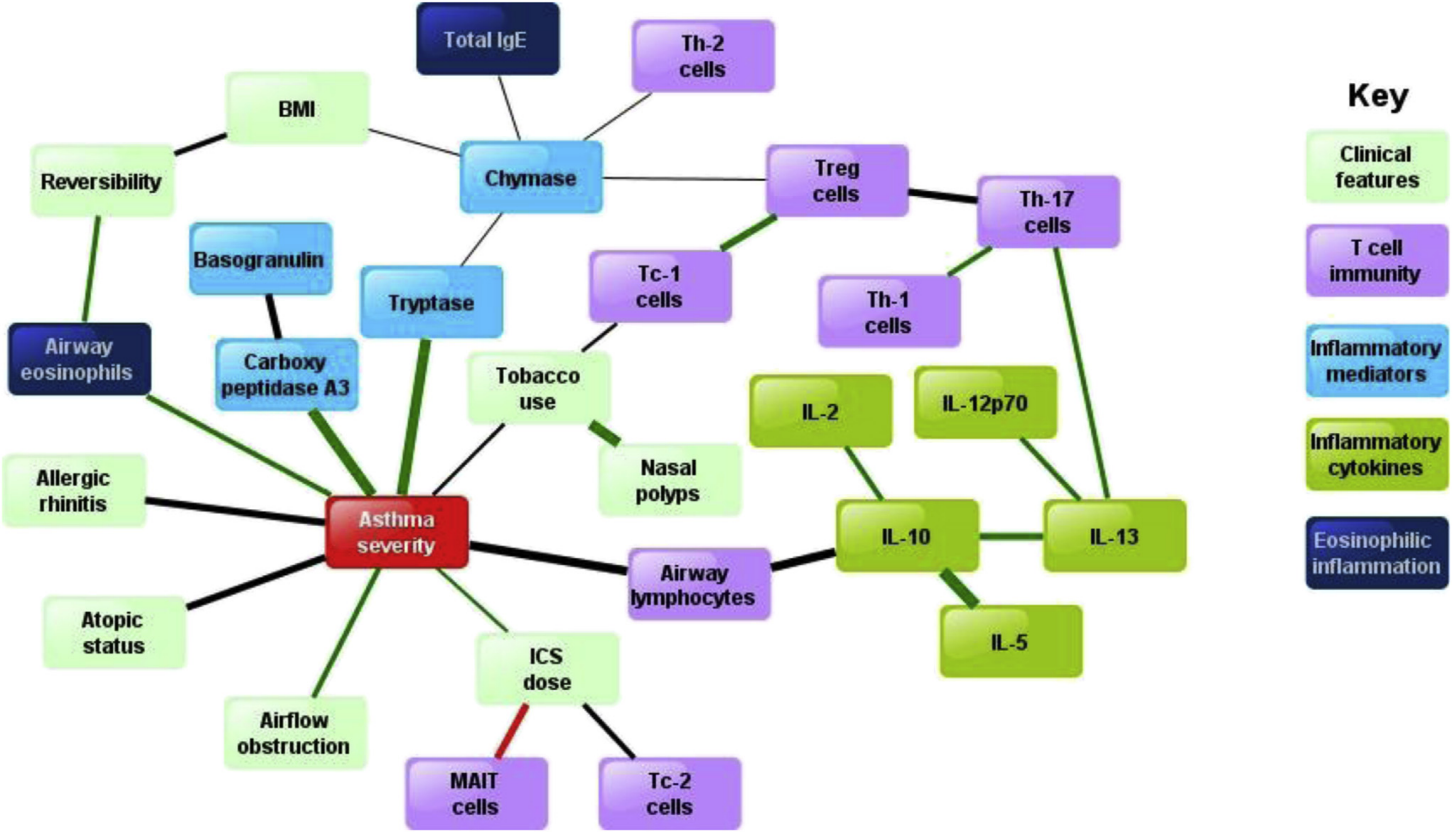
Bayesian belief network showing the strongest interactions between pathobiologic parameters across a range of clinical severities of asthma or health. Nodes without strong interactions are excluded. Line thickness represents strength of interaction (Euclidean distance). Line colors: *green*, positive associations; *red*, negative associations; *black*, nonlinear associations. Asthma severity is based on overall physician's assessment at enrollment (see Table E1). *BMI*, Body mass index; *T*_*C*_*1*, CD8^+^IFN-γ^+^ T cells; *T*_*C*_*2*, CD8^+^IL-13^+^ T cells.

Application of BNA to pathobiologic and clinical features in relation to asthma severity showed complex nonlinear associations (Fig 3). Five nodes (eNO, IL-17, IFN-γ, neutrophils, and vitamin D) without strong interactions with other parameters were not connected and therefore remained outside the network. The asthma severity node was strongly connected with mast cell mediators, tryptase, chymase, and carboxypeptidase A3 and with IL-13– and IFN-γ–secreting CD8^+^ cytotoxic T cells (T_C_2 and T_C_1 cells, respectively). There was a strong negative association between MAIT cell frequencies and ICS use that, in turn, was positively associated with asthma severity.

**Fig 4:**
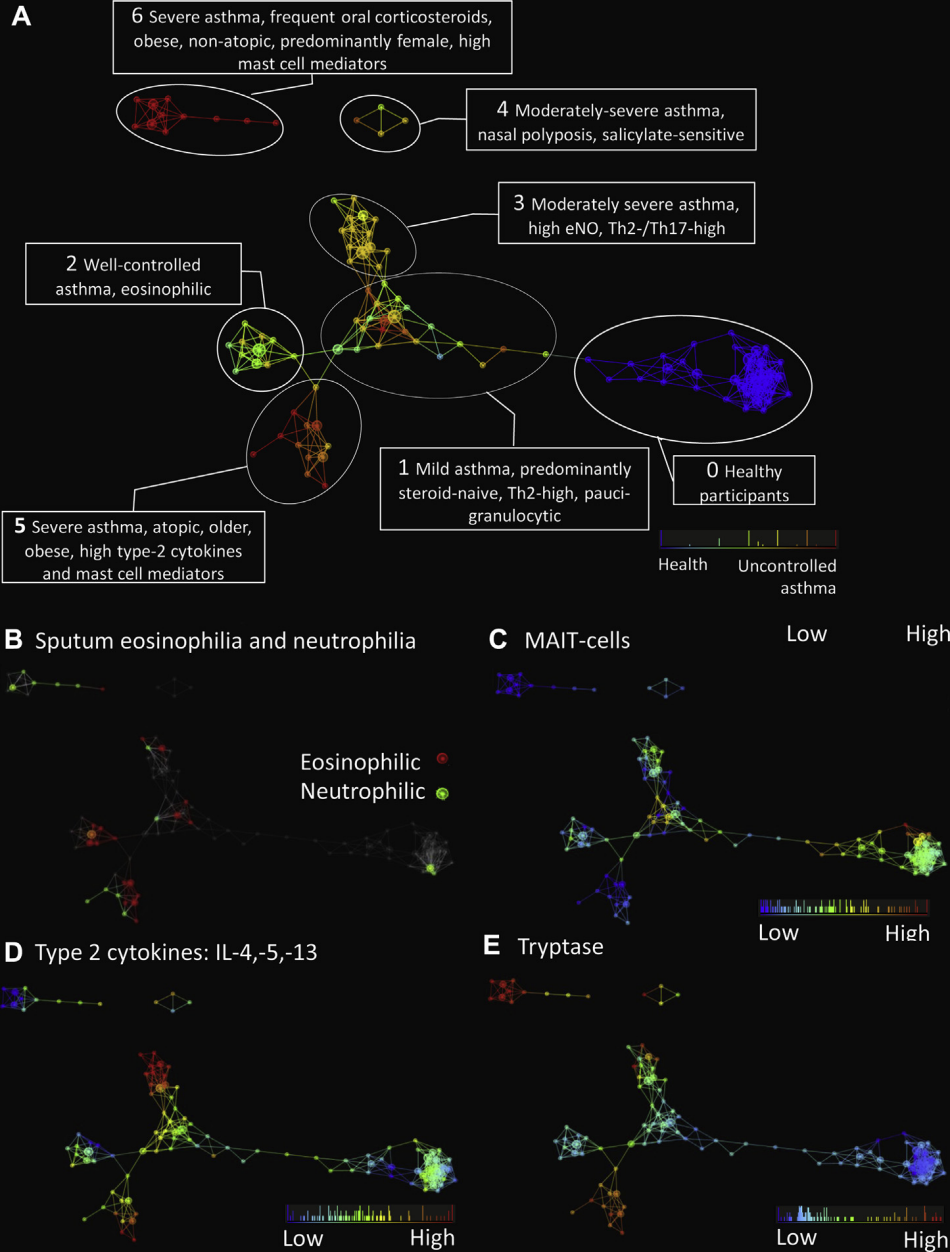
**A,** Multidimensional clinicopathobiologic clusters in asthmatic patients and healthy subjects. Topological network analysis of clinical and pathobiologic features generates 1 healthy *(blue)* and 6 distinct clinicopathobiologic asthma clusters (1-6). The network is colored by disease severity (GINA classification), with patients with the most severe disease in red and patients with the milder forms in varying shades of *orange, yellow*, and *green*. **B,** The same network as Fig 4, *A*, overlaid with distribution of neutrophilic (sputum neutrophils >61%, *green*) or eosinophilic (sputum eosinophils >3%, *red*) asthma. **C,** Frequencies of MAIT cells. **D,** The network is colored based on average concentrations of the type 2 cytokines IL-4, IL-5, and IL-13 in serum, sputum, and BAL fluid. **E,** The network is colored based on concentrations of mast cell tryptase in sputum and BAL fluid. In Fig 4, *B-E*, the colors represent concentrations or frequencies, ranging from low *(blue)* to high *(red)* concentrations. The TDA used 62 subjects with most complete data. The variance normalized Euclidean metric was used. The lenses used were principal and secondary singular value decomposition (resolution, 32; gain, 4.0/3.5×; equalized). Node size is proportional to the number of subjects in the node.

**Fig 5:**
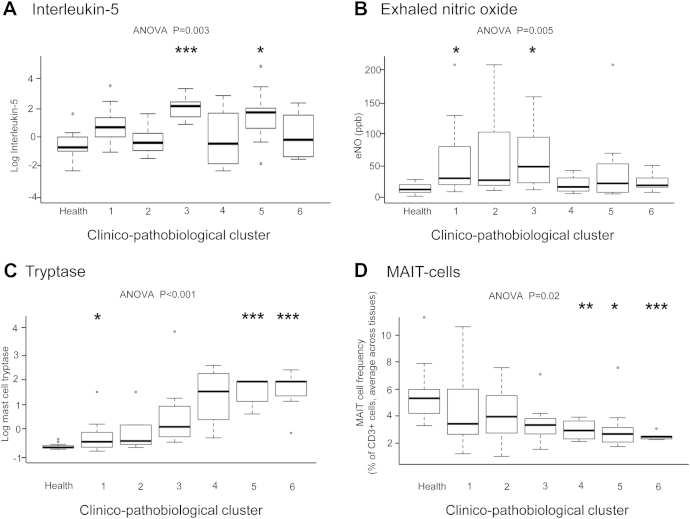
Analyses generated from the clinicopathobiologic TDA network in Fig 4 show concentrations of IL-5 averaged across serum, sputum, and BAL fluid **(A)**; eNO concentrations **(B)**; mast cell tryptase levels in BAL fluid and sputum **(C)**; and MAIT cells in blood, sputum, BAL fluid, and bronchial biopsy specimens **(D)**. *Box and whisker plots* show medians, IQRs, and ranges. Statistical tests indicate 1-way ANOVA, with *post hoc t* tests compared with healthy subjects by using the Bonferroni correction. *P < .05, **P < .01, and ***P < .001.

To look further for novel associations between clinical and pathobiologic features, we applied TDA to all acquired clinical and pathobiologic data (Figs 4 and 5 and see Table E4 in this article's Online Repository at www.jacionline.org). Data were treated as composite averages for T-cell subsets (across blood, sputum, BAL fluid, and biopsy specimens), cytokines and eosinophils (across blood, sputum, and BAL fluid), neutrophils, macrophages, lymphocytes, mast cell mediators, and basogranulin (across sputum and BAL fluid). One healthy and 6 asthma clusters were identified.

The TDA-derived cluster 1, comprising predominantly patients with mild atopic asthma, had (compared with other asthmatic patients) better lung function, lower Asthma Control Questionnaire (ACQ)^27^ scores (mean, 0.88), and lower severity, as assessed by a physician on enrollment or based on Global Initiative for Asthma (GINA) criteria,^20^ and patients were mostly not receiving ICSs. They had increased IL-13–secreting T_H_2 cell numbers and lower IL-13 and tryptase levels and were predominantly paucigranulocytic (sputum neutrophils, ≤61%; eosinophils, ≤3%; Fig 4, *B*, and see Table E4).

Cluster 2 consisted of patients with well-controlled asthma (mean ACQ score, 0.5), with little evidence of inflammation (the only abnormality being eosinophilia) and lower frequencies of Treg cells and IFN-γ–secreting CD8^+^ T cells (Fig 4).

Cluster 3 consisted of patients with moderately severe (defined by enrollment criteria) and partially controlled asthma (based on GINA criteria^20^) despite ICSs; they had the highest bronchodilator reversibility and eNO levels. Their pathobiologic profile consisted of type 2 inflammation, with the highest levels of IL-5 and IL-13 and high frequencies of IL-13−secreting T_H_2 cells in bronchial biopsy specimens (Fig 5, *A*, and see Figs E10 to E12 in this article's Online Repository at www.jacionline.org) but also other T-cell subsets, T_H_1 cells, T_H_17 cells, and Treg cells.

Cluster 4 was a small group with later-onset (mean, 28 years) moderately severe disease based on a physician's assessment, nasal polyposis, salicylate sensitivity, and low IL-17 levels.

Cluster 5 asthmatic patients were older (mean age, 50 years), with high BMI (mean, 32.6 kg/m^2^), poor lung function, high symptom scores (mean ACQ score, 2.1), and high treatment requirements (predominantly GINA step 4-5 and a mean of 1500 μg/d beclomethasone dipropionate equivalent). Their pathobiologic profile was high type 2 cytokine levels (IL-5 and IL-13), IL-13–secreting CD8^+^ T (T_C_2) cells (see Fig E13, *D*, in this article's Online Repository at www.jacionline.org), and high tryptase, chymase, and carboxypeptidase A3 levels (Figs 4 and 5, *C*, and see Figs E10 to E12 in this article's Online Repository). However, when compared with cluster 3 (also type 2 cytokine high), cluster 5 had fewer Treg cells and higher ICS use (mean difference, 1250 μg/d).

Cluster 6 was predominantly female, obese (mean BMI, 35 kg/m^2^), and nonatopic, with salicylate sensitivity and later onset (mean age, 25 years). They were the most severe cluster based on GINA classification, physician's assessment, symptom scores (mean ACQ score, 3.2), and lung function (mean prebronchodilator FEV_1_, 62%), despite high-dose ICSs (see Fig E12, *B*, in this article's Online Repository at www.jacionline.org) and, frequently (50% of the group), maintenance oral corticosteroids (mean, 14 mg/d prednisolone). Their key pathobiologic features were high carboxypeptidase A3 levels and profound MAIT cell deficiency (Figs 4, E, and 5, *D*, and see Fig E9, *D*). They also had low T_C_1, T_H_17, and IL-13−secreting T_H_2 cell numbers (see Fig E9, *A*) but increased tryptase (Figs 4, E, and 5, *C*) and chymase levels. This cluster contained a higher proportion of participants who could be classified as cluster 5, as described by the Severe Asthma Research Program^9^ (see Fig E13, *B*) and the obese, noneosinophilic cluster reported by Haldar et al.^10^

The majority of participants with sputum neutrophilia, which was defined as neutrophil numbers of greater than 61%,^28^ were in clusters 5 and 6, whereas eosinophilic asthma was distributed across clusters 1, 2, 3, and 5 but not clusters 4 or 6 (Fig 4, *B*).

## Discussion

Asthma is a common disease with a clinical severity that ranges from mild forms controlled with β_2_-agonists alone or low doses of ICSs to very severe forms requiring high doses of ICSs and oral corticosteroids and, increasingly, biologics, such as the anti-IgE mAb omalizumab.^29^ In this study comprehensive analysis of T cells, granulocytes, cytokines, and mast cell mediators across the airway lumen, mucosa, and blood compartments pointed to their relative roles within the asthma syndrome that have not been recognized before: reduced MAIT cell frequencies as a striking feature that is related to asthma severity, reduced Treg cell frequencies in severe disease, and increased mast cell mediator levels in patients with severe disease, which is consistent with corticosteroid-insensitive mast cell activation. This study shows that the asthma spectrum can be broken down into several multidimensional clusters defined by combined clinical parameters and underlying mechanisms (pathobiology), which provides proof of concept for endotyping asthma for better understanding of its mechanisms and more focused drug development.

Original descriptions of asthma pathobiology^3^ suggested a key role for T_H_2 mechanisms. Consistent with this concept, when all the asthmatic patients in this study were compared as a group with healthy participants, the most significant asthma discriminators were airway eosinophilia and higher levels of mast cell mediators (carboxypeptidase A3, chymase, and tryptase), IL-5, IL-13, eNO, and serum IgE but lower IFN-γ levels, a pathobiologic profile classically associated with T_H_2 inflammation (Fig 1, *A*, and see Fig E11). The application of BNA showed high connectivity between the asthma severity node and nodes for mast cell mediators and IL-13– and IFN-γ–secreting CD8^+^ cytotoxic T cells (T_C_2 and T_C_1 cells, respectively) and a strong negative association between MAIT cell frequencies and asthma severity and ICS consumption. Applying the recently developed TDA method^21,22^ to the same data set showed complex multidimensional clusters (ie, possible endotypes defined by a combination of clinicopathobiologic features).^8,11^ The advantage of TDA over standard clustering methodologies is that it provides geometric representations of complex and multidimensional data sets that reveal and stratify distinct subgroups.^22^ It combines features of standard statistical methods, such as singular value decompositions and similarity metrics, to construct a network that clusters most similar data points into nodes. A node in a TDA network represents a group of most similar data points (in this case ≥2 subjects who are similar in multiple dimensions). Each node can be joined to the next node if they share common data points (ie, subjects). This allows a natural continuous network when the phenomenon is not disjointed.^21^ TDA can deal with both linear and nonlinear associations and identifies significant subgroups in a data-driven manner, allowing for finer stratification.^21^ Furthermore, TDA is sensitive to both large- and small-scale patterns that other techniques, such as clustering and multidimensional scaling, often do not detect because they sometimes obscure geometric features captured by using topological methods. Hierarchical clustering cannot easily identify these subgroups because it tends to separate points that might in fact be close in the data.^21^

The finding of clinicopathobiologic clusters in the data set in this study should improve our understanding of asthma and inform drug development. Overexpression of the T_H_2 cytokine network in cluster 1 is similar to the original reports in corticosteroid-naive asthmatic patients, highlighting the role of type 2 mechanisms in asthmatic patients.^3^ Clusters 3 and 5 share many clinical features, including atopy, allergic rhinitis, and emotion-related symptoms (see this article's Online Repository). Both are characterized by type 2 inflammation, with the highest levels of IL-5, IL-13, and IL-10, suggesting that these clusters reflect asthma endotypes that might be particularly suitable for biologics, such as mepolizumab^30^ and lebrikizumab,^31^ that currently use indirect biomarkers (ie, eosinophil counts and serum periostin levels) to select patients to maximize clinical efficacy. However, important differences between these 2 clusters were identified: higher Treg cell frequencies in cluster 3 might explain their lower corticosteroid requirements, whereas lower IL-13–secreting T_H_2 cell frequencies and higher tryptase levels in cluster 5 suggest distinct, steroid-insensitive mechanisms.^32^ It should be noted that we stained only for IL-13, and therefore we cannot exclude an increase in T_H_2 cells secreting IL-4 or IL-5 in the more severe asthma clusters. We observed some differences between patterns of cytokine secretion in sputum and BAL fluid, which might arise because BAL samples the distal airways and alveoli, whereas sputum measurement reflects changes in more proximal airways.^33^

The strong association between asthma severity and mast cell mediator levels in clusters 5 and 6 suggests that severe asthma is a disease in which mast cell activation plays an important role. Our data add to evidence implicating mast cells in patients with severe asthma, providing additional confirmation that should stimulate the development of drugs that target mast cells. Brightling et al^6^ have described increased numbers of tryptase-positive mast cells infiltrating the airway smooth muscle in patients with mild and severe^34^ asthma in numbers that correlate with airway hyperresponsiveness.^6,18^ In the Severe Asthma Research Program Balzar et al^16^ reported that severe asthma was associated with an increase in numbers of bronchial mast cells staining positive for both tryptase and chymase and with BAL concentrations of PGD_2_, a lipid mediator associated with mast cells and shown to increase after allergen challenge.^5^

The current study adds to the evidence^5,16,35^ that mast cell activation is insensitive to corticosteroids and suggests that patients with severe asthma, in whom mast cell mediator levels are increased, can be stratified further by clinical features, such as atopic status and also by evidence of type 2 cytokine–mediated mechanisms in cluster 5 but not cluster 6. We speculate that the anti-IgE antibody omalizumab might exhibit some of its beneficial effects in patients with severe atopic asthma through inhibition of IgE-mediated mast cell activation. This finding might yield a prognostic biomarker for this biologic, which is currently missing, and could extend the indication for omalizumab to nonatopic asthma, in which a preliminary trial has suggested clinical efficacy.^36^

Several asthma studies have reported increased IL-17 levels,^37,38^ but this study found only limited evidence for T_H_17 cells and none for γδ-17 cells during either a period of clinical stability or an exacerbation. This is consistent with the findings of a recent trial in which the anti–IL-17 receptor A mAb brodalumab had no effect on symptoms or lung function in patients with moderate-to-severe asthma.^39^ However, we observed associations between IL-17 concentrations and levels of traditional type 2 biomarkers (airways eosinophils and serum IgE) that have not been reported before because IL-17 has mainly been implicated in neutrophilic inflammation in asthmatic patients.^40^

Our study also identified, for the first time, reduced numbers of CD3^+^CD4^+^FOXP3^+^ Treg cells in patients with severe asthma. In human subjects some upregulation of the nuclear transcription factor FOXP3 has been observed in nonsuppressive T cells on T-cell receptor stimulation.^41^ Although we were not able to further validate the identity of these FOXP3^+^ T_H_ cells as Treg cells with additional surface markers, we observed low rates of spontaneous T-cell activation, suggesting that activated T cells will comprise only a small proportion of the CD3^+^CD4^+^FOXP^+^ T cells enumerated.

An important finding in this study is the striking deficiency of MAIT cells in both the circulation and lungs, which correlated strongly with clinical severity. To our knowledge, MAIT cells have not yet been studied in any airways disease. This study suggests that they are more abundant than invariant natural killer T cells,^15^ comprising up to 10% of blood and airway T cells. Their marked evolutionary conservation implies an important role in immunity.^42,43^ MAIT cells are the most abundant T-cell subset able to detect and kill bacteria-infected cells. Recent animal models of bacterial airways infection indicate their critical role in lung host defense.^42,44^ We found MAIT cell frequencies to be associated with serum vitamin D3 concentrations and in pilot data could be suppressed by 1 week of treatment with prednisolone (see Fig E14 in this article's Online Repository at www.jacionline.org). The lack of a significant deficiency of MAIT cells in BAL fluid might result from low peripheral deposition of ICSs in the more distal airways and alveolar compartments sampled by means of lavage.^33^ Their deficiency in patients with severe asthma, whether primary or resulting from chronic corticosteroid use, can contribute to increased susceptibility to bacterial infection recognized in patients with severe asthma^45,46^ and to changes in the airway microbiome^47^ and might thus effect asthma pathology.^44^

In summary, this study sheds light on previously unreported observations in asthma in relation to disease severity. The observation of clusters composed of clinical and pathobiologic parameters will need to be reproduced before these clusters can be accepted as novel endotypes of asthma. However, this paves the way for future asthma studies in large patient cohorts, such as the Severe Asthma Research Program^9^ and Unbiased BIOmarkers in PREDiction of respiratory disease outcomes (U-BIOPRED),^48^ in which distinct asthma endotypes could be identified and subsequently validated, allowing translation to clinical trials and routine clinical practice.

**Key messages**

• We provide proof of concept for a powerful new analytic approach to defining multidimensional clinical and pathobiologic clusters: TDA. This underlines the role of mast cells in 2 distinct subgroups of patients with severe asthma characterized by the presence or absence of type 2 responses.
• Evidence supporting a role for T_H_17 cells in patients with severe asthma is limited.
• We describe a striking deficiency of mucosal-associated T cells, as well as a mild reduction in Treg cell numbers, in patients with severe asthma.

## Figures and Tables

**Table I:** Clinical characteristics of participants

Parameters	Healthy control subjects	Patients with mild asthma	Patients with moderate asthma	Patients with severe asthma
No.	24	15	23	22
Demographics				
Sex (M/F), no. (%)	14 (58)/10 (42)	8 (53)/7 (47)	10 (43)/13 (57)	8 (36)/14 (64)
Age (y), median (range)	28 (20-65)	26 (21-64)	36 (21-56)	53 (23-67)
Pulmonary function				
FEV_1_ (% predicted)	108 (105-113)	88 (86-103)	99 (86-107)	65 (49-82)
FEV_1_ reversibility (%)	3.3 (1.8-7.4)	13 (11-19)	10 (2.2-17)	13 (2.6-25)
PEFR (% predicted)	108 (97-116)	98 (89-107)	95 (85-100)	70 (53-82)
PEFR variability (%)	0 (0-11)	17 (10-25)	22 (17-32)	17 (12-24)
PD_20_ (mg methacholine)	Negative	0.19 (0.050-0.79)	0.25 (0.063-0.73)	Not done
eNO (ppb [at 50 L/s])	16 (11-21)	53 (27-107)	26 (15-51)	20 (13-38)
Clinical				
Atopy (positive skin test result, Y/N), no. (%)	0 (0)/24 (100)	15 (100)/0 (0)	20 (87)/3 (13)	15 (68)/7 (32)
No. of positive skin test results to allergens	0 (NA)	6 (4-7)	3 (2-5)	3.5 (0-5.3)
Peripheral eosinophil count (10^9^/L)	0.1 (0.1-0.2)	0.2 (0.1-0.6)	0.2 (0.15-0.3)	0.2 (0.1-0.3)
Total IgE (IU/mL)	26 (10-61)	172 (21-451)	105 (35-188)	84 (31-669)
Body mass index (kg/m^2^)	24.4 (22.5-28.1)	23.6 (22.7-26.5)	25.3 (23.3-30.9)	31.0 (27.1-40.9)
Smoking status				
Never smoker, no. (%)	21 (88)	14 (93)	19 (83)	17 (77)
Former smoker, no. (% [mean pack-years])	3 (13 [4.2])	1 (7 [6.7])	4 (17 [5.8])	4 (18 [26])
Current smoker, no. (% [mean pack-years])	0 (0)	0 (0)	0 (0)	1 (5 [49])
Duration of asthma (y)	NA	18 (15-26)	22 (9-27)	36 (21-49)
ACQ score	NA	0.60 (0.43-1.3)	1.0 (0.60-1.4)	2.8 (2.2-3.5)
GINA level of control, no. (%)				
Controlled	NA	8 (53)	5 (22)	0 (0)
Partly controlled	NA	6 (40)	15 (65)	2 (9.5)
Uncontrolled	NA	1 (6.7)	3 (13)	19 (90)
Treatment				
Inhaled steroids	No	No	Yes	Yes
Dose (equivalent μg BDP)	NA	NA	400 (200-400)	1600 (1280-2000)
Maintenance oral corticosteroids (Y/N), no. (%)	No	No	No	6 (27)/16 (73)
Mean dose if taken (mg prednisolone/d)				11
Short-acting β-agonist (Y/N), no. (%)	No	Yes	Yes	Yes
Long-acting β-agonist (Y/N), no. (%)	No	No	10 (43)/13 (57)	22 (100)/0 (0)
Leukotriene receptor antagonist (Y/N), no. (%)	No	No	1 (4)/22 (96)	15 (68)/7 (32)
Step on GINA treatment algorithm	NA	1	2-3	4-5
Inflammatory subtype, no. (%)				
Total with valid data	16	13	18	21
Neutrophilic	4 (25)	2 (15)	2 (11)	10 (48)
Eosinophilic	1 (6.3)	3 (23)	3 (17)	6 (29)
Mixed granulocytic	0 (0)	0 (0)	0 (0)	1 (4.8)
Paucigranulocytic	11 (69)	8 (62)	13 (72)	4 (19)
Sputum cell differential (%)				
Macrophages	52 (31-66)	49 (35-64)	47 (30-62)	30 (19-43)
Neutrophils	31 (11-65)	34 (22-54)	33 (16-56)	61 (32-76)
Epithelial	3.6 (2.0-24)	4.3 (1.7-10)	4.1 (1.1-21)	2.9 (0-7.8)
Eosinophils	0.38 (0-0.94)	1.5 (0.75-1.8)	0.75 (0.25-1.5)	0.69 (0-6.1)
Lymphocytes	0.1 (0-0.75)	0.3 (0-0.75)	0 (0-0.68)	0.0 (0-0.25)
BAL cell differential (%)				
Macrophages	84 (74-89)	70 (60-80)	81 (73-89)	72 (46-94)
Neutrophils	2.5 (1.0-5.9)	2.5 (1.6-4.8)	3.5 (1.8-6.4)	6.5 (1.4-29)
Epithelial cells	9.9 (3.9-18)	21 (13-35)	11 (5.6-19)	8.7 (3.3-11)
Eosinophils	0.25 (0.0-0.56)	2.0 (0.75-3.6)	1.0 (0-3.0)	0.1 (0-1.6)
Lymphocytes	1.4 (0.94-2.4)	1.5 (0.38-3.0)	1.3 (0.5-2.3)	1 (0-1.6)
Relevant comorbidities, no. (%)				
Allergic rhinitis	0 (0)	12 (80)	11 (58)	10 (46)
Nasal polyps	0 (0)	0 (0)	1 (5.3)	5 (23)
Eczema	3 (13)	7 (47)	6 (32)	4 (19)
Bronchiectasis	0 (0)	0 (0)	1 (5.3)	1 (4.5)

Values are medians with IQRs, unless stated otherwise. The inflammatory subtype is based on sputum differentials using the following cut points: neutrophilic, >61%; eosinophilic, >3%. Percentages given are derived from subjects with valid data. *BDP*, Beclomethasone dipropionate; *NA*, not available; *PEFR*, peak expiratory flow rate.

## Supplementary data

Online Repository Text

Fig E1

Fig E2

Fig E3

Fig E4

Fig E5

Fig E6

Fig E7

Fig E8

Fig E9

Fig E10

Fig E11

Fig E12

Fig E13

Fig E14
