# Supplementary material for: Innate and adaptive T cells in asthmatic patients: Relationship to severity and disease mechanisms
Source: J Allergy Clin Immunol. 2015 Aug;136(2):323–33. doi: 10.1016/j.jaci.2015.01.014 (PMC4534770; doi:10.1016/j.jaci.2015.01.014)
Supplement: Online Repository Text [file mmc1.docx]

**SUPPLEMENTARY APPENDIX**

Table of Contents

Supplementary information on methods 4

Preparation of airway samples 4

Flow cytometry 4

Confirmation of MAIT-cell receptor sequence 6

Measurement of mast cell proteases 7

Measurement of basogranulin 7

Measurement of cytokines 8

Topological data analysis 8

Data elaboration for TDA 9

Analysis of seasonal variation of MAIT-cells 10

Supplementary Results 11

Presentation of clinical classification in the main text and the supplementary appendix 11

Further analysis of data from the complete cohort without imputation 11

Longitudinal cohort 12

MAIT-cells are deficient in asthma, modulated by corticosteroids and associated with vitamin D levels*.* 13

Analysis of mast cell mediators stratified by physician assessment of severity 15

Additional results from TDA applied to clinico-pathobiologic parameters 15

Comparison of TDA with clusters identified by other authors 17

Figure Legends 19

Figure E1. Flow diagram showing study design and recruitment. 19

Figure E2. Representative flow cytometry plots 19

Figure E3. Major CD4+ T cell subsets stratified by disease severity 19

Figure E4. T-cell cytokines in bronchoalveolar lavage fluid and sputum supernatant 20

Figure E5 IL-17 secreting γδ T cells in health and asthma in peripheral blood and bronchoalveolar lavage 21

Figure E6 T cell frequencies in peripheral blood and sputum during acute viral infection-induced exacerbations. 21

Figure E7 Peripheral blood Th17 response according to whether experienced an exacerbation. 22

Figure E8. Clinical correlates of mucosal associated invariant T-cells frequencies 22

Figure E9. Additional pathobiologic features of clinico-pathobiologic clusters 22

Figure E10. CD4+ T cell subsets in bronchial biopsies according to clinico-pathobiologic clusters 23

Figure E11. Inflammatory mediators in bronchoalveolar lavage (A,B,D,E) and sputum (C,F) in health and asthma 23

Figure E12. Analyses generated from clinico-pathologic TDA network shown in Figure 4. 24

Figure E13. Other features amongst clinico-pathobiologic clusters 24

Figure E14. Peripheral blood MAIT-cell frequencies over the course of the year and after oral corticosteroids 25

Tables 26

Table E1. Criteria used to attribute physician assessed severity of asthma at the time of enrolment 26

Table E2. Definition of terms used in the topological data analysis 28

Table E3. Parameters included in the topological data analyses 32

Table E4. Descriptions of clinico-pathobiologic clusters 34

References 38

Title: Innate and adaptive T cells in asthma: relationship to severity and disease mechanisms

Timothy SC Hinks MD^1,2^, Xiaoying Zhou PhD^1^, Karl J Staples PhD^1^, Borislav D Dimitrov MD^2,3^, Alexander Manta PhD^4^, Tanya Petrossian PhD^5^, Pek Y Lum PhD^5^, Caroline G Smith RN^3^, Jon A Ward BSc^1,2^, Peter H Howarth MD^1,2^, Andrew F Walls PhD^1^, Stephan D Gadola MD^1,6^, Ratko Djukanović MD^1,2^

1. Clinical and Experimental Sciences, University of Southampton Faculty of Medicine, Sir Henry Wellcome Laboratories, Southampton University Hospital, Southampton, SO16 6YD, UK

2. Southampton NIHR Respiratory Biomedical Research Unit, Southampton University Hospital, Southampton, SO16 6YD, UK

3. Primary Care and Population Sciences, University of Southampton Faculty of Medicine, Southampton University Hospital, Southampton, SO16 6YD, UK

4. MantaMatics UG, Dompfaffenweg 23a, 82538 Geretsried, Germany

5. Ayasdi Inc., 636 Ramona Street, Palo Alto, CA 94301, USA

6. Novartis Institute of Biomedical Research, Novartis Basel WSJ-386, Switzerland

Contact:

r.djukanovic@soton.ac.uk

Tel: +44 2381 204195

Fax: +44 2380 511761

## Supplementary information on methods

### Preparation of airway samples

Sputum induction was performed according to the recommendations of the European Respiratory Society^1^ and the samples were immediately processed with dithioerythritol to separate cells from the fluid phase of the sputum, as previously described^2^. Fibreoptic bronchoscopy for bronchoalveolar lavage (BAL) and bronchial biopsy was performed in accordance with the recommendations of the American Thoracic Society.^3^ For flow cytometry cells were processed either immediately (for analysis of MAIT-cells) or cultured overnight for analysis of other subsets. Prior to antibody staining, bronchial biopsies were dispersed with collagenase I (Sigma) reconstituted in RPMI 1640 without L-glutamine (Sigma) at 1 mg/ml for 1 hour at 37°C.^2^

### Flow cytometry

MAIT-cells were identified in peripheral blood mononuclear cells (PBMC), sputum, BAL and biopsies by flow cytometry for surface markers, using a nine-color FACS Aria^TM^ cell sorter (BD Biosciences, Oxford, UK). Live cells were identified using LIVE/DEAD® Fixable Violet Dead Cell Stain for 405 nm excitation (L34955, Invitrogen) according to the manufacturer’s instructions. Cells were resuspended and washed in fluorescence activated cell sorting (FACS) buffer (0.5% (w/v) Bovine Serum Albumin, 2 mM EDTA in Phosphate Buffered Saline [pH 7·4]) then stained with fluorochrome conjugated antibodies in 110 μl FACS buffer for 30 min at 4°C. Doublets were excluded using pulse-width and pulse-area and side scatter properties.^2^ MAIT-cells were identified by the following monoclonal antibodies in combination with anti-CD3, anti-CD4, anti-CD8 monoclonal antibodies (BD Biosciences): PE–conjugated anti-Vα7.2 (clone 3C10, Biolegend, San Diego, CA) and FITC-conjugated anti-CD161 monoclonal antibody (BD Biosciences). γδ T-cells were identified by anti-γδ TCR-FITC (BD Biosciences).

For intracellular cytokine analysis that allowed identification of Th17, Th1 and IL-13-secreting Th2 T-cells, whole bronchial biopsies were cultured overnight in AIM V® serum free medium (Gibco, Life Technologies, Paisley, UK) supplemented with: 0.5 μg/ml Fungizone, 2 mM L-glutamine, 1 mM sodium pyruvate, 100 μg/ml streptomycin,100 U/ml penicillin (all Gibco) and 0.004% (v/v) 2-mercaptoethanol (Agilent Technologies UK, Stockport, UK) at 37°C in the presence of 5% CO_2_, as previously described by our group.^2^ Cell aliquots obtained by sputum induction or bronchoscopy, were resuspended in AIM V® at a concentration of 1x10^6^ cells/ml, and cultured overnight in the same manner. The following day, samples were stimulated for 5 h with 25 ng/ml phorbol 12-myristate 13-acetate (PMA) and 500 ng/ml ionomycin (both Sigma, Gillingham, UK) in the presence of 2 μM monensin (eBioscience, Hatfield, UK). Paired samples were left unstimulated, without PMA, ionomycin or monensin, as negative controls and for FOXP3 staining. Bronchial biopsies were then dispersed with collagenase I. Cells were stained using 1 μl LIVE/DEAD® Fixable Violet for 30 min, washed again and fix-permeabilized with eBioscience fixation/permeabilization reagents according to the manufacturer’s instructions. CD4+ and CD8+ T-cell subsets were identified by intracellular staining for 45 min with the following monoclonal antibodies: anti-IL-13-FITC (R&D Systems, Abingdon, UK), anti-IL-17A-PE, anti-interferon(IFN)-γ-allophycocyanin (APC), anti-Tumor Necrosis Factor (TNF)-α-APC, anti-FOXP3-APC (all eBioscience), followed by cytometry as described above. Representative flow cytometry plots are presented in Figure E2.

### Confirmation of MAIT-cell receptor sequence

In order to validate whether cells identified by dual staining for TCR-Vα7.2 and CD161 were indeed MAIT cells, the identity of the TCR was confirmed by PCR. Briefly, live CD3+Vα7.2+ CD161+ cells were sorted directly *ex vivo* from peripheral blood into sorting media and cell lines (clones) established. After 2-3 weeks culture *ex vivo* these clones were again stained and Vα7.2+CD161+ cells were sorted. RNA was extracted from using TRIzol® LS Reagent (LifeTechnologies) and chloroform (Sigma), reverse transcribed using SuperScriptTM III reverse transcriptase (Invitrogen, Paisley, UK) and amplified using reverse-transcription polymerase chain reaction performed using BioTaq DNA Polymerase (Gentaur, Brussels, Belgium). The reaction mixture, 50 μl total, contained 1 μl of cDNA, 5 μl 10x NH4 buffer, 2.5 μl of 50 mM MgCl2, 1μl of 10mM dNTP, 0.5 μl of BioTaq DNA polymerase, 1 μl of forward primer, 1 μl of reverse primer and 39 μl of ddH_2_0. Amplifications were performed on the Mastercycler (Eppendorf, Stevenage, UK) with the following cycling parameters: 96 ̊C for 1 minute pre-incubation, then 96 ̊C for 30 s denaturing, then 65 ̊C for 30 s annealing, then 72 ̊C for 2 min elongation; repeated for 45 cycles and finished with a 10 min extension at 72 ̊C. Primers (MWG Biotech AG, Ebersberg, Germany) were designed to span the canonical Vα7.2-Jα33 rearrangement of the CDR3α region of the TCRα chain.^4^

1. TCR Vα7.2

5’-ATA TAT CAT ATG GGA CAA AAC ATT GAC CAG-3’ forward

2. TCR Jα33

5’-GCT TTA TAA TTA GCT TGG TCC CAG C-3’ reverse

10 μl of reaction product premixed with dye were loaded into wells of a 1% (w/v) agarose (Melford, Chelsworth, UK) gel in Tris/Borate/EDTA buffer containing 10 μl per 100 ml of Nancy-520 dye (Sigma) and electrophoresed at 80 volts for 60 min, before imaging under ultraviolet light. A clear product of appropriate length was identified in these clones and in a positive control (Jα33 cDNA) but not in a non-MAIT iNKT clone (negative control), confirming that cells identified according to their surface staining did indeed express the canonical Vα7.2-Jα33 TCR rearrangement.

### Measurement of mast cell proteases

Various combinations of polyclonal and monoclonal antibodies prepared against tryptase, chymase or carboxypeptidase A3 were investigated in a sandwich ELISA development. Immunoglobulins were purified from hybridoma culture supernatants by ammonium sulphate precipitation^5^ followed by protein G affinity chromatography (GE healthcare). For use as detecting antibodies immunoglobulins were biotinylated by biotinamidocaproate N-hydroxysuccinimide ester (Sigma).

The ELISA for tryptase was performed as previously described,^5^ with rabbit antiserum as coating antibody and monoclonal antibody AA5 for detection. Proteases purified from human skin^6^ were employed as assay standards. Chymase and carboxypeptidase A3 were measured by sandwich ELISA using monoclonal antibodies developed in-house.

### Measurement of basogranulin

Basogranulin levels were determined by a dot blotting procedure with specific monoclonal antibody BB1 essentially as described previously.^7^ A lysate of peripheral blood basophils purified using MACS Basophil Isolation Kit (Milteny Biotec, Bisley, UK) served as the standard.

### Measurement of cytokines

Cytokines were measured by multiplex enzyme-linked electrochemiluminescent assays (Meso Scale Discovery, Gaithersburg, MD) in serum, sputum supernatant and BAL. For cytokine assays of BAL samples, BAL supernatants were first concentrated 45-fold by centrifugal dialysis using ‘Vivaspin 6’ concentrators (Sartorius Stedim Biotech GmbH, Göettingen, Germany) at 2000 g for 4.5 h at 4°C.

### Topological data analysis

TDA was performed using variance normalized Euclidean distance as a distance-metric. The Normalized Euclidean distance between two points takes into account that each column in the data set could have significantly different variance. The Normalized Euclidean (or Variance Normalized Euclidean, VNE) distance between two points X and Y is given by:


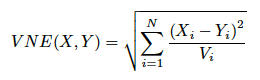


Where V_i_ is the variance associated with each column *i* and is given by


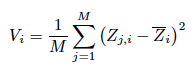


And Z*_i_* is the mean of column *i* and is given by


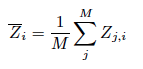


TDA also used two filter functions: principal and secondary metric singular value decomposition. These filter lenses maps data into a Euclidean space using the rows of the distance matrix as the coordinates and then performs principal component analysis. The data is subsequently projected via multi-dimensional scaling (MDS) by finding an embedding for *X*, *Z*:


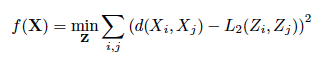


### Data elaboration for TDA

TDA analyses used data from the 62 participants with the most complete datasets. A wide range of parameters, in multiple compartments (blood, sputum, BAL and bronchial biopsies), some requiring invasive testing, were acquired which meant that nearly 16% of data were missing values. However, as each cytokine, mediator or T-cell subset had been measured in paired compartment samples from each participant, it was possible to minimize the impact of missing data by generating for each parameter a composite average metric across each tissue. Composite variables have been used by others^8^ and were necessary as both Bayesian network analysis and TDA are sensitive to missing data, with potential to create subgroups that are an artefact, to an extent determined by the fact that there are many missing values.

First, missing data were imputed for each individual parameter using the average value specific to each compartment and disease severity subgroup, the severity being defined by clinical and physiologic criteria defined before the study began (see table E1). Data were normalized by dividing each parameter by the group mean and a single composite average value was then generated for each parameter using the average across each compartment, thereby creating a matrix of 62 participants and 52 rows (26 pathobiologic parameters, 26 clinical parameters). Composite averages were obtained for T-cell subsets (from blood, sputum, BAL and biopsies), cytokines, eosinophils (from blood, sputum, BAL), neutrophils, macrophages, lymphocytes, mast cell mediators, basogranulin (from sputum and BAL). Comparisons between multiple groups used one-way analysis of variance (ANOVA), followed by post-hoc *t* tests with Bonferroni correction for multiple comparisons: using as reference category the healthy controls. Composite variables used in the TDA are defined in Table E2. Parameters which are used to generate the network are listed in Table E3.

Throughout the main paper, results presented are only for these composite averages. However, it was also possible to perform additional analyses for each parameter according to each specific tissue using the full dataset from all 84 participants without the need for any imputation. This approach has the advantage of determining how processes differ between distinct tissue compartments. For instance in comparison with BAL, sputum is considered as more representative of processes occurring in the more proximal rather than distal airways. These analyses are presented in this supplementary appendix in addition to further analyses using composite variables (see the results section below).

### Analysis of seasonal variation of MAIT-cells

A cyclical pattern throughout the entire period during which the study was performed was suspected to explain the variations of MAIT-cell frequencies over time; this was analyzed using non-linear regression on log-transformed data using a standard sine function, as follows:

Ln (MAIT frequency) = 0·0795+0.6024*sin ((2π*[Seasonal quarter]/4)+78.27)

Likewise a cyclical pattern in serum vitamin D3 concentrations was analyzed as follows:

Ln (vitamin D3) = 3.865+0.4804*sin ((2π*[Seasonal quarter]/4)+78.27)

The quarters began on 1^st^ January and ended on 31^st^ December.

## Supplementary Results

### Presentation of clinical classification in the main text and the supplementary appendix

Initial clinical classification of all recruited participants was performed using the criteria agreed by the authors prior to the start of the analysis (see Table E1). All participants were stratified as healthy, mild (steroid-naïve), moderate or severe based on the criteria applied at enrolment. Participants with asthma were also classified by the GINA criteria^9^ and characterized further using the Asthma Control Questionnaire^10^ and the GINA control criteria^9^ to enable readers to interpret the results of this study in relation to widely used classification methods.

The application of TDA to the combined clinical and pathobiologic parameters created 6 multi-dimensional clinico-pathobiologic asthma clusters which are named as cluster-1 to cluster-6, in increasing order of overall clinical severity according to GINA criteria.

### Further analysis of data from the complete cohort without imputation

IL-17 has usually been hypothesized to be implicated in neutrophilic inflammation in asthma.^11-13^ However we observed an increase in IL-17 in a subset of patients with mild, steroid naïve asthma, which did not correlate with an increase in Th17 cell frequencies. An alternative cellular source of IL-17 might be the respiratory epithelium. Consistent with this, it was noted that BAL IL-17 concentrations were elevated exclusively in participants in whom the same BAL contained sheets of bronchial epithelium. When participants were dichotomized by the upper limit of normal range (97.5^th^ percentile in health) for BAL epithelial cell frequencies, median BAL IL-17 concentrations are 4.0 fold higher in participants with abnormally high epithelial cell counts (0.023 pg/ml (IQR, 0.018-0.076)) than other participants (0.0057 (IQR, 0.0024-0.0090), P<0.001)(Figure E4C). IL-17 has been implicated in inflammation of the upper airway epithelium.^14^ Inflammation of the lower airway epithelium is known to cause loss of epithelial integrity,^15^ due to disruption of tight junctions which may be promoted by IL-17.^16^ Therefore BAL IL-17 may be associated with epithelial fragility.

### Longitudinal cohort

Whilst our data suggest that Th17 cells did not play a role in stable asthma, we considered the possibility there might be a dysregulation of the dynamics of the Th17/Treg response during acute, viral exacerbations of asthma, which are known to be associated with neutrophilic inflammation.^17^ We therefore measured the dynamics of the Th17, Th1, IL-13-secreting Th2 and Treg subsets in peripheral blood and sputum in 26 frequently-exacerbating asthmatics during a naturally occurring upper respiratory tract infection. We observed no significant changes in T cell frequencies in peripheral blood, and only a 1.8-fold increase in mean sputum Th17 cells from 6.2% at baseline to 11% at symptom day 7 (P=0.034)(Figure E6). Furthermore we observed no significant difference in the area under of curves of PBMC Th17 response between subjects whose upper respiratory tract infection was complicated by an acute asthma exacerbation (mean 12.5, (95%CI, 7.95-17.2)) and those where it was not (mean 7.59, (95%CI, 3.93-11.2), P=0.2, Figure E7). Together these data suggest that Th17 and γδT-17 cells do not play a significant role in human asthma.

### **MAIT-cells are deficient in asthma, modulated by corticosteroids and associated with vitamin D levels***.*

Having observed a deficiency of TCR Vα7.2+ CD161+ mucosal associated invariant T (MAIT) cells in asthma we sought to elucidate potential causes. We first considered whether this might be related to age, in view of the slightly higher age of the participants with more severe asthma. An additional cohort of older healthy control participants (median age of 53 years, IQR 48-57, *n*=12) was, therefore, recruited and analysis of blood MAIT-cell frequencies showed no difference in this additional cohort when compared with younger healthy participants (median age 27, IQR 24-34, *n=*15, P=0.4) (data not shown).

Next we considered whether this deficiency might be related to the therapeutic use of corticosteroids and, therefore, performed two open-label studies with inhaled and oral corticosteroids. No significant change in MAIT-cell frequencies was seen in blood or sputum of 12 steroid-naïve mild asthmatic participants after a 7-day course of low dose (200 micrograms twice daily) of ultrafine particle beclometasone dipropionate (Qvar) (data not shown). By contrast, there was a significant (P=0.03), 23% decrease in median peripheral blood MAIT-cell frequencies after 7 days of prednisolone given at a dose of 20 milligrams once daily (median 1.63% (1.1-4.4%) pre-treatment compared with median 1.26% (0.92-1.9%) post treatment)(Figure E14A). Furthermore, this steroid-induced modulation of T-cell frequencies was specific to MAIT-cells as it was not observed with a non-MAIT T-cell subset (median 1.91% (1.4-2.2%) pre-treatment compared with median 1.88% (1.5-2.3%) post treatment, P>0.05, Figure E14B). This suggests that the observed MAIT-cell deficiency may have been caused by chronic use of corticosteroids, but further in-depth studies are required to confirm and elucidate further the nature of this apparent cause-effect relationship.

Murine studies have suggested that vitamin D3 is important for the development and function of iNKT-cells,^18^ and this has been linked to experimental airways hyper-reactivity (AHR) in mice.^19^ Therefore, we sought to identify first whether there was a seasonal pattern in MAIT-cell frequencies given the known seasonal variation in vitamin D levels.^20^ This showed a highly significant association between blood MAIT-cell frequencies and the season in which blood samples were taken (ANOVA on log-transformed data, P<0.001), with peak frequencies in August and a nadir in February (Figure E14C), consistent with an association with sunlight exposure. Regression analysis suggested that seasonal variation explained 16% of the variance in MAIT-cell frequencies (R^2^=0.16). This effect was specific to MAIT-cells as it was not observed with a non-MAIT-cell population (Vα7.2+CD161- cells, ANOVA P>0.05, data not shown) and was not due to corticosteroid therapy as it was observed in both the healthy and mild-asthmatic participants who were not on corticosteroids. We then completed the investigation by measuring serum vitamin D3 concentrations in serum samples paired with the blood MAIT-cell samples using mass spectrometry. This showed significant association (Pearson’s R^2^=0.28, P=0.01) between serum vitamin D and blood MAIT-cell frequencies, and regression analysis showed that 28% of the variability in MAIT-cell frequencies is attributable to variations in serum vitamin D3 (Figure E14D, ANOVA P<0.0001 with *post hoc* Dunnett’s ***P<0.001 when compared with January-March).

### Analysis of mast cell mediators stratified by physician assessment of severity

Data on airway mast cell mediators are presented in the main text stratified according to the clusters identified by TDA. In addition, data from the full dataset are presented in Figure E11, comparing asthma with health (Figure E11 A-C) and stratified according to physician assessment of severity at baseline (Figure E11 D-F). In asthma there was a significant increase in the mast cell mediators tryptase (median 5.4 ng/ml (4.6-9.4) in asthma, median 4.3 ng/ml (4.0-4.8) in health, P=0.003) and carboxypeptidase A3 (median 0.35 ng/ml (0.065-1.0) in asthma, median 0.057 ng/ml (0.016-0.097) in health, P=0.001), but not chymase (median 2.4 ng/ml (0.0-5.4) in asthma, median 1.1 ng/ml (0.0-3.1) in health, P=0.4) in bronchoalveolar lavage, compared with health. When stratified by disease severity carboxypeptidase was significantly elevated in mild and severe groups, with a similar, but non-significant tendency in the moderate group. Likewise carboxypeptidase was significantly elevated in the mild and moderate groups with a similar but non-significant tendency in the severe group. By contrast in sputum asthma was associated with a significant increase only in chymase (median 1.4 ng/ml (0.0-10) in asthma, median 0.0 ng/ml (0.0-0.46) in health, P=0.04), and this was significant for the severe group.

### Additional results from TDA applied to clinico-pathobiologic parameters

In addition to the results presented in the main paper, a number of additional features are apparent within the dataset. It is important to note that, after ANOVA, *post-hoc* analysis used a very conservative approach to correct for multiple comparisons, applying Bonferroni correction. Nevertheless, these analyses should be seen as exploratory, to be seen as hypothesis generating rather than hypothesis testing.

Figures E9A and B provide evidence that type-2 inflammation (as defined by IL-13-secreting Th2-cells, and the cytokines IL-5 and IL-13) is not a uniform feature of asthma. Flow cytometry revealed the highest frequencies of Th2-cells in the bronchial biopsy tissue from cluster-3 (Figure E9A). The type-2 cytokine, IL-13 (composite average across serum, sputum and BAL) was also most elevated in cluster-3, but was also significantly elevated compared cluster-5. As the asthmatics in cluster-5 were on high dose ICS (mean 1500 mcg bdp equivalent) and had low Th2 frequencies, this suggests that IL-5 and IL-13 were from a different cellular origin and that the mechanisms regulating their production are steroid-insensitive. Possible sources include innate lymphoid cells,^21^ IL-13 secreting CD8+ (Tc2) cells, mast cells, basophils, eosinophils^22,23^ and macrophages.^24^

The doses of inhaled corticosteroid (ICS) were higher in the severe cluster-5 and cluster-6 (Figure E12). Long-term maintenance oral corticosteroid use was almost exclusive to group F, with a median dose of 14 mg/day oral prednisolone in the 50% of participants who were taking this drug.

Additional differences amongst clusters are shown in Table E4. Atopy (defined as a positive skin prick test to at least 1 common aeroallergen) was common in clusters 1,3,4 and 5, but significantly less common in cluster 6 (P=0.01). Likewise, allergic rhinitis was less common in cluster-4 and cluster-6, whilst nasal polyposis was observed only in the three more severe clusters-4 to 6. As groups 3 and 5 had in common the pathobiologic feature of high type-2 cytokines – the highest levels of IL-5, IL-13 and IL-10 – these groups were compared. When compared with other asthmatic clusters, both cluster-3 and cluster-5 had higher rates of atopy (P<0.05), and symptoms triggered by exercise (P<0.05) or by emotion (P<0.001). However, cluster-5 differed from cluster-3 at a pathobiologic level as group 5 had significantly higher tryptase (P=0.02) and lower frequencies of Treg (P=0.02).

Evidence suggests that mast cells may respond to stimuli such as changes in the osmotic environment associated with exercise. We, therefore, compared pathobiologic features in participants with and without exercise induced asthma. We observed that the only pathobiologic feature associated with exercise-induced asthma was elevated levels of tryptase (composite from BAL and sputum, KS P=0.004), with a similar trend for carboxypeptidase A3 (P=0.052) but not for chymase (data not shown).

### Comparison of TDA with clusters identified by other authors

In order to compare how the clinico-pathobiologic clusters in the current study compare with previously reported endotypes, each participant in the current study was classified according to either the hierarchical decision tree used by the Severe Asthma Research Program (SARP)^8^ or by a best fit approach to the endotypes described by Haldar *et al^25^* and Lötvall *et al^26^* and then mapped these on to the TDA network. In general there was no close agreement with descriptions by others, which may not be surprising as these had focused primarily on clinical features.

In our topological analysis the location of each subject within the network is determined to some extent by every single clinical and pathobiologic parameter; the relative influence of each being represented within Table E4 by the KS scores. Nonetheless, the formation of some clusters appeared to be influenced predominantly by homogeneous clinical features, such as cluster-2 – significantly characterized by low symptomatology (see Table E4) without any dominant influence from a single immunological parameter. Conversely other clusters were influenced more by a constellation of pathobiologic parameters. For instance cluster-3 is distinguished by higher frequencies of T cell subsets or higher concentrations of T cell cytokines, implying perhaps a distinct pathobiologic process of T cell activation underlying this cluster. The composition of the other clusters is in each case driven by a mixture of interacting clinical and pathobiologic parameters.

Nonetheless, participants in the clinico-pathobiologic group 6 were significantly more likely (75% of group) than other participants (15% of group) to be classified by SARP as ‘cluster 5’, i.e. severe, later onset, female predominant (*t* test P=0.002)(Figure E13B). Similarly, participants in cluster-6 tended to be in the ‘obese non-eosinophilic’ cluster described by Haldar, although this association did not reach statistical significance (63% of group v 26%, *t* test P=0.054). Lötvall *et al.* specified several other putative clusters from their literature review,^26^ which included ‘severe, late onset, hypereosinophilic asthma’, and ‘aspirin-sensitive asthma’; the latter cluster mapped on to cluster 4 and cluster-6 (both 50% of group v 11% *t* test P=0.005) (Figure E13C).

## Figure Legends

### Figure E1. Flow diagram showing study design and recruitment.

### Figure E2. Representative flow cytometry plots

Typical flow cytometry plots for T-helper cell subsets measured by intracellular cytokine staining. Doublets were excluded by gating on forward scatter-area (pulse area) versus forward scatter-width (pulse-width). Dead cells were excluded by their increased uptake of Violet LIVE/DEAD® Fixable. T cells were identified by side scatter profile and surface staining for CD3-PE-Cy7. T helper cells were identified by staining for CD4-PerCP-Cy5.5 (PBMC, BAL, sputum) or by lack of staining for CD8-APC-Cy7 (bronchial biopsies). Gates for IL-17, IFN-γ and IL-13 were set on controls comprising unstimulated cells using the same stains. Treg were identified by intracellular staining for FoxP3. As FOXP3 is not significantly expressed in CD4- cells this gate was set by the level amongst CD4-ve cells, such that the P1 gate contained ≤0.5% of CD3+ cells, as shown.

### Figure E3. Major CD4+ T cell subsets stratified by disease severity

(a) Ratios of CD3+4+ T cells expressing IL-13 (Th2 cells), to those expressing IFN-γ (Th1 cells) and (b) frequencies of CD3+4+ T cells expressing IFN-γ (Th1 cells) in PBMC, sputum, BAL and bronchial biopsies measured by intracellular cytokine staining and flow cytometry. Results are expressed as a percentage of live CD3+CD4+ T cells, or for bronchial biopsies, as a percentage of CD3+8- T cells. Medians are shown as horizontal lines. Left hand column of panels shows healthy controls compared with asthmatics, and P values are from Mann-Whitney U tests. Right three columns of panels show the same data stratified by disease severity (defined at enrolment) where P values are from Kruskal-Wallis tests and are given where P<0.05. Significance post hoc by Dunn’s compared with health: *P<0.05, ** P<0.01. Abbreviations: BAL, bronchoalveolar-lavage, PBMC, peripheral blood mononuclear cell.

### Figure E4. T-cell cytokines in bronchoalveolar lavage fluid and sputum supernatant

Cytokines were measured in duplicate in bronchoalveolar lavage (BAL) (A) and sputum (B) from 60 individuals (18 healthy controls, 12 mild, 16 moderate and 14 severe asthmatics). (C) BAL IL-17 was associated with allergic rhinitis, airways eosinophilia, serum IgE and the extent of epithelial shedding (see results in supplementary appendix for the method used to assess shedding). Cytokines were measured by multiplex enzyme-linked electrochemiluminescent assay using the Meso Scale Discovery platform. BAL samples were first concentrated 45-fold by centrifugal dialysis. Log-transformed data were compared by ANOVA (where significant, the P value is given at the top of each figure). Significance *post -hoc* by Dunn’s compared with health: *P<0.05, ** P<0.01, *** P<0.001.

Abbreviations: BAL, bronchoalveolar-lavage; LOD (---), limit of detection; *r*_s_, Spearman’s correlation coefficient.

### Figure E5 IL-17 secreting γδ T cells in health and asthma in peripheral blood and bronchoalveolar lavage

Frequencies of γδ T cells secreting IL-17 as a proportion of total γδ T cells in peripheral blood and in bronchoalveolar lavage measured by intracellular cytokine staining and flow cytometry. Groups are compared by Mann-Whitney (two groups) or Kruskal-Wallis tests (multiple groups). Frequencies of γδ T cells were too low for accurate assessment in sputum or bronchial biopsies. Abbreviations: BAL, bronchoalveolar-lavage, PBMC, peripheral blood mononuclear cell.

### Figure E6 T cell frequencies in peripheral blood and sputum during acute viral infection-induced exacerbations.

Frequencies of CD4+ T cells in (A) peripheral blood and (B) induced sputum during exacerbations caused by acute upper respiratory tract infections measured by intracellular cytokine staining and flow cytometry on samples processed directly *ex vivo*. Day 0, baseline screening visit (n=14); day 4, symptom day 4 (n=13); day 7, symptom day 7 (n=14). Plots show means ± 95% confidence intervals. In general data are not paired. Mean frequencies of sputum Th17 cells increased significantly 1.8-fold from 6.2% at baseline to 11% at symptom day 7. (C) T cell frequencies in paired samples of cryopreserved peripheral blood taken from 26 individuals at day 0, baseline screening visit, at day 1 and days 4, 7, 10, 13, 17 (each ±1 day) and day 30-35, showing the dynamics of the T cell response with greater temporal resolution.

### Figure E7 Peripheral blood Th17 response according to whether experienced an exacerbation.

(A) Peripheral blood Th17 cell frequencies measured by flow cytometry on cryopreserved samples plotted over time and stratified by whether subjects experienced an exacerbation of their asthma defined as a 0.5 point fall in asthma control questionnaire between screening and symptom day 7 (n=16 exacerbated, n=10 did not). Plots show mean ±95% confidence interval.

(B) Plot of areas under the curve (AUC) for peripheral Th17 response over time, stratified by whether subjects experienced an exacerbation. Areas under curves were calculated from the frequency of Th17 cells at each of 8 time-points and then compared using unpaired *t* tests according to the method of Matthews^27^.

### Figure E8. Clinical correlates of mucosal associated invariant T-cells frequencies

Frequencies of Vα7.2+CD161+ (MAIT)-cells in peripheral blood (A,C-F) and sputum (B) are negatively correlated with (A,B) dose of inhaled corticosteroids, (D) asthma control questionnaire score, (E) GINA level of control, (F) step on GINA treatment algorithm and positively correlated with FEV1 (as a percentage of predicted). *r_s_*, Spearman’s correlation coefficient.

### Figure E9. Additional pathobiologic features of clinico-pathobiologic clusters

Analyses were generated from the clinico-pathobiologic TDA network shown in Figure 4 from the main text. (A) Frequencies of CD3+8- IL-13+ (Th2) cells measured by flow cytometry in bronchial biopsies are elevated specifically in the mild-moderate clusters A to C. (B) Concentrations of interleukin-13 averaged across serum, sputum and bronchoalveolar lavage. C) and D) Average concentrations of mast cell (C) chymase (ng/ml) and (D) carboxypeptidase A3 in BAL and sputum are markedly elevated in clusters E and F (severe, steroid-refractory disease). Box and whisker plots show medians, interquartile ranges and ranges. Statistical tests: one way ANOVA, with *post-hoc* *t* tests when compared with health, using Bonferroni correction (*P<0.05, ** P<0.01, *** P<0.001).

### Figure E10. CD4+ T cell subsets in bronchial biopsies according to clinico-pathobiologic clusters

Analyses were generated from the clinico-pathobiologic TDA network shown in Figure 4 from the main text. Frequencies of (A) CD3+8- IL-17+ (Th17) cells, (B) CD3+8-IFN-γ (Th1) cells, (C) CD3+8-IL-13+ (Th2) cells and (D) CD3+8-FoxP3+ (Treg) measured by flow cytometry in bronchial biopsies. Box and whisker plots show medians, interquartile ranges and ranges. Statistical tests: one way ANOVA, with post-hoc t tests when compared with health, using Bonferroni correction (*P<0.05, ** P<0.01, *** P<0.001).

Figure E11. Inflammatory mediators in bronchoalveolar lavage (A,B,D,E) and sputum (C,F) in health and asthma

Concentrations of tryptase (A and D), (B) carboxypeptidase A3 (B and E) in BAL, and sputum of chymase (c and f). Differences are compared by Mann Whitney tests (two groups). P values are for Kruskal-Wallis tests (multiple groups) with *post hoc* Dunn’s compared with health: * P<0.05, ** P<0.01.

### Figure E12. Analyses generated from clinico-pathologic TDA network shown in Figure 4.

(A) Asthma severity differs widely between different clinico-pathologic subgroups: named from 1 to 6 in increasing severity using GINA classification of health (0), controlled asthma (1), partially controlled asthma (2) and uncontrolled asthma (3). (B) Dose of inhaled corticosteroids (ICS) in micrograms beclometasone equivalent (mcg bdp) per day. Box and whisker plots show medians, interquartile ranges and ranges. Statistical tests: one way ANOVA, with *post hoc t* tests compared with health, using Bonferroni correction (*P<0.05, ** P<0.01, *** P<0.001).

### Figure E13. Other features amongst clinico-pathobiologic clusters

(A) Clinico-pathobiologic network colored according to average IL-13-secreting Th2 cell frequencies in blood, sputum, BAL and biopsies (red, high frequencies; blue, low frequencies). (B) Distribution of asthmatics fulfilling the criteria of ‘cohort 5’ defined by the Severe Asthma Research Program.^8^ (C) Aspirin-sensitive asthma (red: present, blue: absent, green / yellow: mixed nodes containing some subjects with and some without aspirin-sensitivity). (D) Frequencies of IL-13 secreting CD8+ (Tc2) cells in bronchial biopsies; note the close correlation, i.e. co-localization, with sputum eosinophilia.

The TDA used 62 subjects with most complete data; Metric: variance normalized Euclidean; Lenses: principal and secondary singular value decomposition (SVD)(Resolution 32, Gain 4.0/3.5x, Equalized); Node size: proportional to number of individuals in node. Color bars: red high values, blue: low values.

### Figure E14. Peripheral blood MAIT-cell frequencies over the course of the year and after oral corticosteroids

(A and B) Frequencies of peripheral blood MAIT (Vα7.2+CD161+) cells and non-MAIT T-cells (Vα7.2+CD161- cells) in 12 moderate asthmatic subjects, usually controlled on inhaled corticosteroids, before and after 7 days of treatment with 20 milligrams of oral prednisolone given once daily. Frequencies are shown as a percentage of live CD3+ T-cells. P values are for paired *t* tests: * P<0.05, ** P<0.01.

(C) Peripheral blood MAIT-cell frequencies and (d) serum vitamin D3 concentrations vary over the course of the year and are highest in the summer months. (c) Log-transformed Vα7.2+CD161+ cell frequencies from healthy and asthmatic subjects according to the quarter in which phlebotomy was performed, with a sinusoidal regression line. R^2^=0.16. (D) Log-transformed vitamin D3 concentrations from healthy and asthmatic subjects according to the quarter in which phlebotomy was performed, with a sinusoidal regression line. R^2^=0.30.

## Tables

### Table E1. Criteria used to attribute physician assessed severity of asthma at the time of enrolment

| **Mild asthma** |
| --- |
| Symptoms < once a day  Nocturnal symptoms < twice a month  • FEV_1_ or PEF ≥80% predicted  Treatment:  • Salbutamol as needed only  Presence of asthma confirmed by methacholine challenge testing. |
| **Moderate asthma** |
| Symptoms < once a day  Nocturnal symptoms <once a week  Asthma control score >1.5  Treatment:  • Salbutamol as needed only  • Low-dose inhaled steroids (<800 mcg beclometasone dipropionate equivalent)  • +/- Long acting beta-2-agonist  Presence of asthma confirmed by methacholine challenge testing. |
| **Severe asthma** |
| Symptoms daily  Nocturnal symptoms >once a week  Daily use of inhaled short-acting ß2-agonist  • FEV_1_ or PEF <80% of predicted or patient’s best  Treatment:  • High-dose inhaled steroids ( ≥800 mcg beclometasone dipropionate equivalent)  • Long acting beta-2-agonist  • +/- frequent or continuous oral corticosteroids |

These criteria^2^ were agreed before the study began and used by T.S.C.H. to assign each participant with asthma to one of 3 categories of disease severity after full clinical and physiologic assessment during screening/enrolment. Where patients did not map on to a single category they were considered on an individual basis to achieve the best possible fit.

### Table E2. Definition of terms used in the topological data analysis

| **Term** | | **Definition** | **Compartments** | **Assay** |
| --- | --- | --- | --- | --- |
| T-cell subsets | |  |  |  |
|  | Th1 | CD3+CD4+IFNγ+ cells | Composite average across blood, sputum, BAL, bronchial biopsy | 9 color fluorescence activated flow cytometry |
|  | Th2 | CD3+CD4+IL-13+ cells |  |  |
|  | Th17 | CD3+CD4+IL-17+ cells |  |  |
|  | Treg | CD3+CD4+FOXP3+ cells |  |  |
|  | Tc1 | CD3+CD8+IFNγ+ cells |  |  |
|  | Tc2 | CD3+CD8+IL-13+ cells |  |  |
|  | MAIT | CD3+Vα7.2+CD161+ (MAIT) cells |  |  |
| Cytokines | |  |  |  |
|  | IFN-γ | Interferon-γ concentrations | Composite average across blood, sputum, BAL | Electrochemi-luminescent assay |
|  | IL-2 | Interleukin-2 concentrations |  |  |
|  | IL-4 | Interleukin-4 concentrations |  |  |
|  | IL-5 | Interleukin-5 concentrations |  |  |
|  | IL-10 | Interleukin-10 concentrations |  |  |
|  | IL-12p70 | Interleukin-12p70 concentrations |  |  |
|  | IL-13 | Interleukin-13 concentrations |  |  |
| Mast cell mediators | |  |  |  |
|  | Chymase | Chymase concentrations | Composite average across sputum, BAL | ELISA |
|  | Carboxypeptidase A3 | Carboxypeptidase A3 concentrations |  |  |
|  | Tryptase | Tryptase concentrations |  |  |
| Inflammatory cells | |  |  |  |
|  | Neutrophils | Neutrophil frequencies | Composite average across sputum, BAL | Cell counts |
|  | Lymphocytes | Lymphocyte frequencies |  |  |
|  | Macrophages | Macrophage frequencies |  |  |
|  | Eosinophils | Eosinophil frequencies | Composite average across blood, sputum, BAL |  |
| Inflammatory subtype | |  |  |  |
|  | Neutrophilic | Sputum subclass: neutrophils >61%^28^ | Sputum | Cell counts |
|  | Eosinophilic | Sputum subclass: sputum eosinophils >3%^29^ | Sputum |  |
|  | Paucigranulocytic | Sputum subclass: neutrophils ≤61%,^28^ sputum eosinophils ≤3% | Sputum |  |
| Other | |  |  |  |
|  | eNO | Exhaled nitric oxide levels (ppb) | Exhaled breath | Niox Mino® at 50mL/s |
|  | IgE | Serum immunoglobulin E | Serum | ELISA |
|  | Vitamin D3 | Vitamin D3 concentration | Serum | Mass spectrometry |
| Clinical | |  |  |  |
|  | BMI | Body Mass Index |  |  |
|  | FEV_1_ | Spirometric forced expiratory volume in 1 second (L), pre-bronchodilator |  |  |
|  | Reversibility | Percentage change in FEV_1_ before and 15 minutes after 500 micrograms nebulized salbutamol |  |  |
|  | GINA severity | Asthma severity according to GINA classification^9^ assigned values:(0) health, (1) controlled asthma, (2) partially controlled asthma, (3) uncontrolled asthma. |  |  |
|  | Physician assessed severity | Asthma severity assessed by a physician (T.S.C.H.) using criteria in Table E1 |  |  |

### Table E3. Parameters included in the topological data analyses

| **Pathologic parameters**^A^ | | | | | | | | | | |
| --- | --- | --- | --- | --- | --- | --- | --- | --- | --- | --- |
| eNO | | IgE | |  |  |  |  | |  |  |
| IL-4 | | IL-5 | | IL-13 | IL-17 | IL-2 | IL-12p70 | | IFN-γ | IL-10 |
| Th17 | | Th1 | | Th2 | Treg | Tc1 | Tc2 | | MAIT |  |
| Macrophages | | | | Lymphocytes | | Neutrophils | | | Eosinophils | |
| Tryptase | | Chymase | | Carboxypeptidase A3 | | Basogranulin | | | Vitamin D3 | |
| **Clinical parameters** | | | | | | | | | | |
| Age | Gender | | Inflammatory subtype | | | ACQ | | BMI | | |
| Age of onset of asthma | | | | | | Atopic status | | | | |
| History of symptoms induced by foods | | | | | | History of smoking (pack years) | | | | |
| History of virus-induced exacerbations | | | | | | History of eczema | | | | |
| History of exercise-induced symptoms | | | | | | History of allergic rhinitis | | | | |
| History of symptoms induced by aerosols | | | | | | History of nasal polyposis | | | | |
| History of symptoms induced by emotion | | | | | | | | | | |
| History of symptoms induced by aeroallergens | | | | | | | | | | |
| History of symptoms induced by changes in air temperature of humidity | | | | | | | | | | |
| History of symptoms induced by salicylates ^B^ | | | | | | | | | | |
| Inhaled corticosteroid dose | | | | | | Oral corticosteroid dose | | | | |
| Use of long acting beta-2 agonists | | | | | | Use of leukotriene receptor antagonists | | | | |
| Peak expiratory flow (percent predicted) | | | | | | | | | | |
| FEV_1_ (pre-bronchodilator) | | | | | | Bronchodilator reversibility | | | | |

^A^Other parameters which incorporate *a priori* assumptions or overlap extensively other parameters were not used to generate the main TDA network: physician assessment of severity, GINA severity, treatment burden (step on GINA treatment algorithm). ^B^ ‘Salicylate’ refers to symptoms induced by either salicylates or non-salicylate non-steroidal anti-inflammatory drugs.

### Table E4. Descriptions of clinico-pathobiologic clusters

| **Endo- type** | | | **Features of cluster ^A^** | **Kolmogorov-Smirnov tests** | | **Comments** |
| --- | --- | --- | --- | --- | --- | --- |
|  | | | | **KS score** | **P value** |  |
| 1 | Clinical | | |  |  |  |
|  |  | Lower asthma severity by enrolment criteria | | 0.643 | 0.0005 | Mostly physician assessed as 'mild' |
|  |  | Lower GINA severity | | 0.482 | 0.02 | Mostly GINA severity 'controlled' |
|  |  | Lower ACQ | | 0.434 | 0.04 | Mean ACQ 0·88 |
|  |  | Lower treatment step (GINA) | | 0.699 | 0.0001 | Mostly GINA treatment step 1 |
|  |  | Lower ICS Dose | | 0.607 | 0.001 | Mostly not on ICS |
|  |  | Higher FEV_1_ (% pred) | | 0.496 | 0.01 | Mostly atopic |
|  |  | Higher PEFR (% pred) | | 0.496 | 0.01 |  |
|  |  | More likely paucigranulocytic sputum | | 0.430 | 0.047 |  |
|  | Pathobiologic | | |  |  |  |
|  |  | Higher Th2 | | 0.485 | 0.02 |  |
|  |  | Lower tryptase | | 0.585 | 0.002 |  |
|  |  | Lower IL-13 | | 0.430 | 0.047 |  |
|  |  | Lower vitamin D3 | | 0.515 | 0.01 |  |
| 2 | Clinical | | |  |  |  |
|  |  | Lower ACQ | | 0.690 | 0.02 | Mean ACQ 0.5 |
|  |  | Lower BMI | | 0.667 | 0.02 |  |
|  |  |  | |  |  |  |
| 3 | Pathobiologic | | |  |  |  |
|  |  | Higher Th17 | | 0.607 | 0.02 | Mostly physician assessed as 'moderate' |
|  |  | Higher Th1 | | 0.585 | 0.03 | Mostly GINA severity 'partially controlled' |
|  |  | Higher IL-5 | | 0.561 | 0.04 | Highest reversibility (mean 18%) |
|  |  | Higher IL-12p70 | | 0.607 | 0.02 | Highest eNO (mean 65 ppb) |
|  |  | Higher IFN-γ | | 0.604 | 0.025 |  |
| 4 | Pathobiologic | | |  |  | Mostly physician assessed as 'moderate' |
|  |  | Lower IL-17 | | 0.861 | 0.02 | Highest age of onset (median 33 years) |
|  |  |  | |  |  | Highest frequency of nasal polyps and salicylate sensitivity ^B^ |
|  |  |  | |  |  |  |
| 5 | Clinical | | |  |  |  |
|  |  | Older age | | 0.556 | 0.03 | Mean age 50 years |
|  |  | Lower PEFR (% pred) | | 0.530 | 0.04 | High BMI (mean 32.6) |
|  |  | Higher treatment step (GINA) | | 0.581 | 0.02 | Mostly GINA treatment step 4/5 |
|  |  | Higher ICS dose | | 0.598 | 0.01 | Mean ICS dose 1500 mcg |
|  | Pathobiologic | | |  |  | High ACQ (mean 2.1) |
|  |  | Higher tryptase | | 0.667 | 0.004 |  |
|  |  | Higher IL-13 | | 0.513 | 0.049 |  |
|  |  | Higher Tc2 | | 0.539 | 0.03 |  |
|  |  | Lower basogranulin | | 0.530 | 0.038 |  |
| 6 | Clinical | | |  |  |  |
|  |  | Higher ACQ | | 0.744 | 0.003 | Highest ACQ (mean 3.2) |
|  |  | Higher treatment step (GINA) | | 0.670 | 0.009 | Mostly GINA treatment step 5 |
|  |  | Higher ICS dose | | 0.693 | 0.006 | Mean ICS dose 1400 mcg |
|  |  | Higher LTRA use | | 0.721 | 0.004 | Highest BMI (mean 35.3) |
|  |  | Higher use of reliever medication | | 0.663 | 0.02 | Mostly female (88%) |
|  |  | Higher age of onset | | 0.556 | 0.047 | High median age of onset 30 |
|  |  | Lower FEV_1_ (% pred) | | 0.647 | 0.01 | Lowest FEV_1_ (mean 62% pred) |
|  | Pathobiologic | | |  |  | High frequency of salicylate sensitivity ^B^ |
|  |  | Higher tryptase | | 0.619 | 0.02 | 50% of group OCS (mean 14mg prednisolone/day) |
|  |  | Higher carboxypeptidase A3 | | 0.571 | 0.04 |  |
|  |  | Lower MAIT | | 0.593 | 0.03 |  |
|  |  | Lower Th2 | | 0.564 | 0.04 |  |
|  |  | Lower Th1 | | 0.622 | 0.02 |  |
|  |  | Lower Th17 | | 0.593 | 0.03 |  |
|  |  | Lower Tc1 | | 0.644 | 0.014 |  |

^A^ Features of each cluster are parameters that differ significantly in Kolmogorov-Smirnov tests between each cluster and a group comprised of the remaining participants. ^B^ ‘Salicylate’ refers to symptoms induced by either salicylates or non-salicylate non-steroidal anti-inflammatory drugs. Abbreviations: ACQ, Seven point Asthma Control Score;^10^ BMI, body mass index; GINA, Global Initiative for Asthma;^9^ ICS, inhaled corticosteroids; OCS, oral corticosteroids. For other terms see table E2.

## References

1. Djukanovic R, Sterk PJ, Fahy JV, Hargreave FE. Standardised methodology of sputum induction and processing. Eur Respir J Suppl 2002;37:1s-2s.

2. Vijayanand P, Seumois G, Pickard C, et al. Invariant natural killer T cells in asthma and chronic obstructive pulmonary disease. N Engl J Med 2007;356:1410-22.

3. Djukanovic R, Wilson JW, Lai CK, Holgate ST, Howarth PH. The safety aspects of fiberoptic bronchoscopy, bronchoalveolar lavage, and endobronchial biopsy in asthma. Am Rev Respir Dis 1991;143:772-7.

4. Porcelli S, Yockey CE, Brenner MB, Balk SP. Analysis of T cell antigen receptor (TCR) expression by human peripheral blood CD4-8- alpha/beta T cells demonstrates preferential use of several V beta genes and an invariant TCR alpha chain. J Exp Med 1993;178:1-16.

5. Buckley MG, Variend S, Walls AF. Elevated serum concentrations of beta-tryptase, but not alpha-tryptase, in Sudden Infant Death Syndrome (SIDS). An investigation of anaphylactic mechanisms. Clin Exp Allergy 2001;31:1696-704.

6. McEuen AR, Walls AF. Purification and characterisation of mast cell tryptase and chymase from human tissues. In: Jones MG, Lympany L, eds. Allergy Methods and Protocols, Methods in Molecular Medicine. Totowa, NJ: Humana Press; 2008:299-317.

7. Mochizuki A, McEuen AR, Buckley MG, Walls AF. The release of basogranulin in response to IgE-dependent and IgE-independent stimuli: validity of basogranulin measurement as an indicator of basophil activation. J Allergy Clin Immunol 2003;112:102-8.

8. Moore WC, Meyers DA, Wenzel SE, et al. Identification of asthma phenotypes using cluster analysis in the Severe Asthma Research Program. Am J Respir Crit Care Med 2010;181:315-23.

9. (GINA) GIfA. Global Strategy for Asthma Management and Prevention2012.

10. Juniper EF, O'Byrne PM, Ferrie PJ, King DR, Roberts JN. Measuring asthma control. Clinic questionnaire or daily diary? Am J Respir Crit Care Med 2000;162:1330-4.

11. Molet S, Hamid Q, Davoine F, et al. IL-17 is increased in asthmatic airways and induces human bronchial fibroblasts to produce cytokines. J Allergy Clin Immunol 2001;108:430-8.

12. Park H, Li Z, Yang XO, et al. A distinct lineage of CD4 T cells regulates tissue inflammation by producing interleukin 17. Nat Immunol 2005;6:1133-41.

13. McKinley L, Alcorn JF, Peterson A, et al. TH17 cells mediate steroid-resistant airway inflammation and airway hyperresponsiveness in mice. J Immunol 2008;181:4089-97.

14. Xu G, Zhang L, Wang DY, et al. Opposing roles of IL-17A and IL-25 in the regulation of TSLP production in human nasal epithelial cells. Allergy 2010;65:581-9.

15. Swindle EJ, Collins JE, Davies DE. Breakdown in epithelial barrier function in patients with asthma: identification of novel therapeutic approaches. J Allergy Clin Immunol 2009;124:23-34; quiz 5-6.

16. Gutowska-Owsiak D, Schaupp AL, Salimi M, et al. IL-17 downregulates filaggrin and affects keratinocyte expression of genes associated with cellular adhesion. Experimental dermatology 2012;21:104-10.

17. Wark PA, Johnston SL, Moric I, Simpson JL, Hensley MJ, Gibson PG. Neutrophil degranulation and cell lysis is associated with clinical severity in virus-induced asthma. Eur Respir J 2002;19:68-75.

18. Yu S, Cantorna MT. Epigenetic reduction in invariant NKT cells following in utero vitamin D deficiency in mice. J Immunol 2011;186:1384-90.

19. Yu S, Zhao J, Cantorna MT. Invariant NKT cell defects in vitamin D receptor knockout mice prevents experimental lung inflammation. J Immunol 2011;187:4907-12.

20. Hypponen E, Power C. Hypovitaminosis D in British adults at age 45 y: nationwide cohort study of dietary and lifestyle predictors. The American journal of clinical nutrition 2007;85:860-8.

21. Brusselle GG, Maes T, Bracke KR. Eosinophils in the Spotlight: Eosinophilic airway inflammation in nonallergic asthma. Nat Med 2013;19:977-9.

22. Voehringer D, Reese TA, Huang X, Shinkai K, Locksley RM. Type 2 immunity is controlled by IL-4/IL-13 expression in hematopoietic non-eosinophil cells of the innate immune system. J Exp Med 2006;203:1435-46.

23. Wynn TA. IL-13 effector functions. Annu Rev Immunol 2003;21:425-56.

24. Kim EY, Battaile JT, Patel AC, et al. Persistent activation of an innate immune response translates respiratory viral infection into chronic lung disease. Nat Med 2008;14:633-40.

25. Haldar P, Pavord ID, Shaw DE, et al. Cluster analysis and clinical asthma phenotypes. Am J Respir Crit Care Med 2008;178:218-24.

26. Lotvall J, Akdis CA, Bacharier LB, et al. Asthma endotypes: a new approach to classification of disease entities within the asthma syndrome. J Allergy Clin Immunol 2011;127:355-60.

27. Matthews JN, Altman DG, Campbell MJ, Royston P. Analysis of serial measurements in medical research. BMJ 1990;300:230-5.

28. Simpson JL, Scott R, Boyle MJ, Gibson PG. Inflammatory subtypes in asthma: assessment and identification using induced sputum. Respirology 2006;11:54-61.

29. Green RH, Pavord I. Stability of inflammatory phenotypes in asthma. Thorax 2012;67:665-7.
