# Supplementary material for: Innate and adaptive T cells in asthmatic patients: Relationship to severity and disease mechanisms
Source: J Allergy Clin Immunol. 2015 Aug;136(2):323–33. doi: 10.1016/j.jaci.2015.01.014 (PMC4534770; doi:10.1016/j.jaci.2015.01.014)
Supplement: Fig E1 [file mmc2.ppt]

## Slide 1
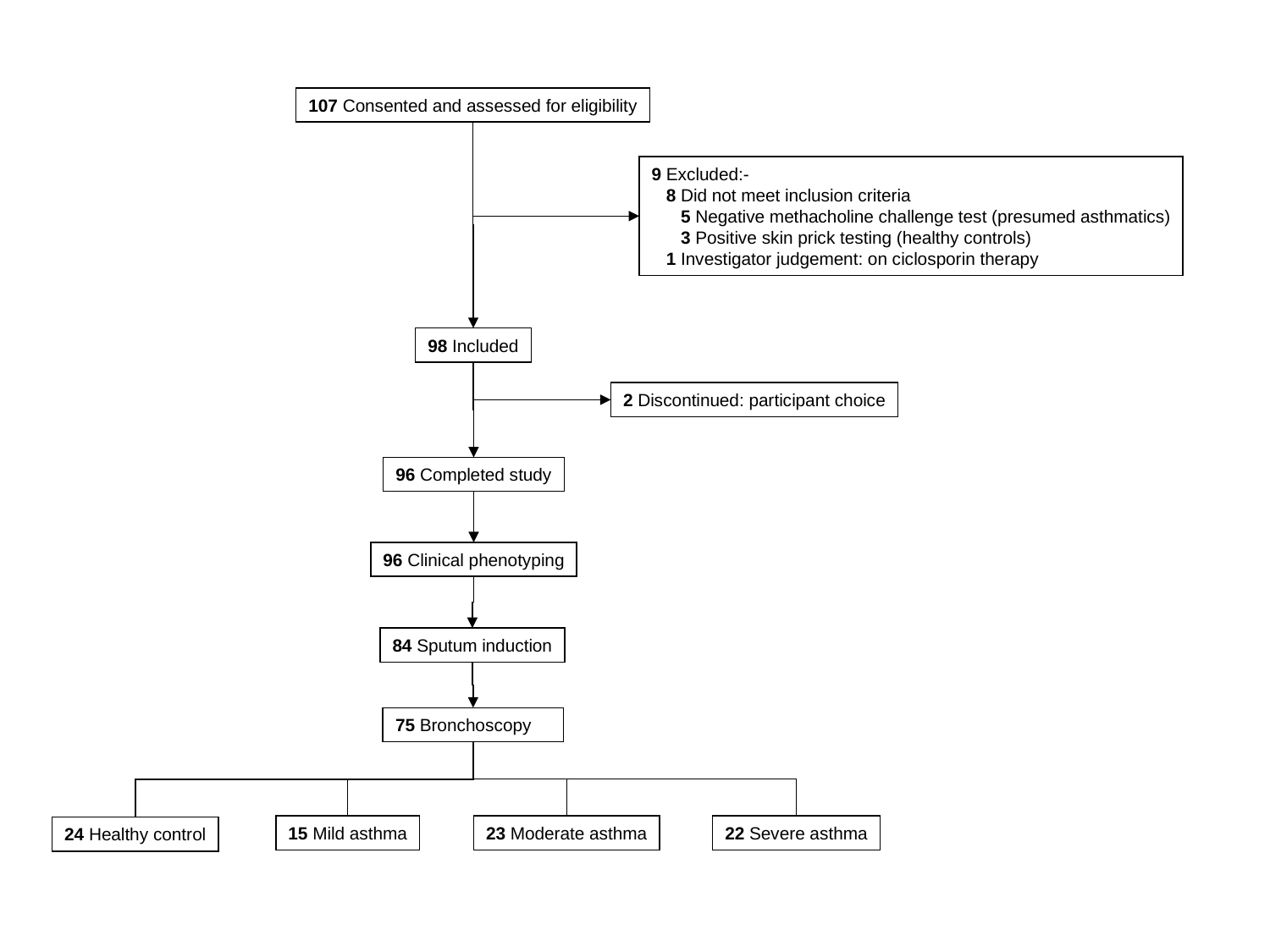

107 Consented and assessed for eligibility
9 Excluded:-
 8 Did not meet inclusion criteria
 5 Negative methacholine challenge test (presumed asthmatics)
 3 Positive skin prick testing (healthy controls)
 1 Investigator judgement: on ciclosporin therapy
98 Included
2 Discontinued: participant choice
96 Completed study
96 Clinical phenotyping
84 Sputum induction
75 Bronchoscopy
15 Mild asthma
23 Moderate asthma
22 Severe asthma
24 Healthy control
