# Supplementary material for: Innate and adaptive T cells in asthmatic patients: Relationship to severity and disease mechanisms
Source: J Allergy Clin Immunol. 2015 Aug;136(2):323–33. doi: 10.1016/j.jaci.2015.01.014 (PMC4534770; doi:10.1016/j.jaci.2015.01.014)
Supplement: Fig E2 [file mmc3.ppt]

## Slide 1
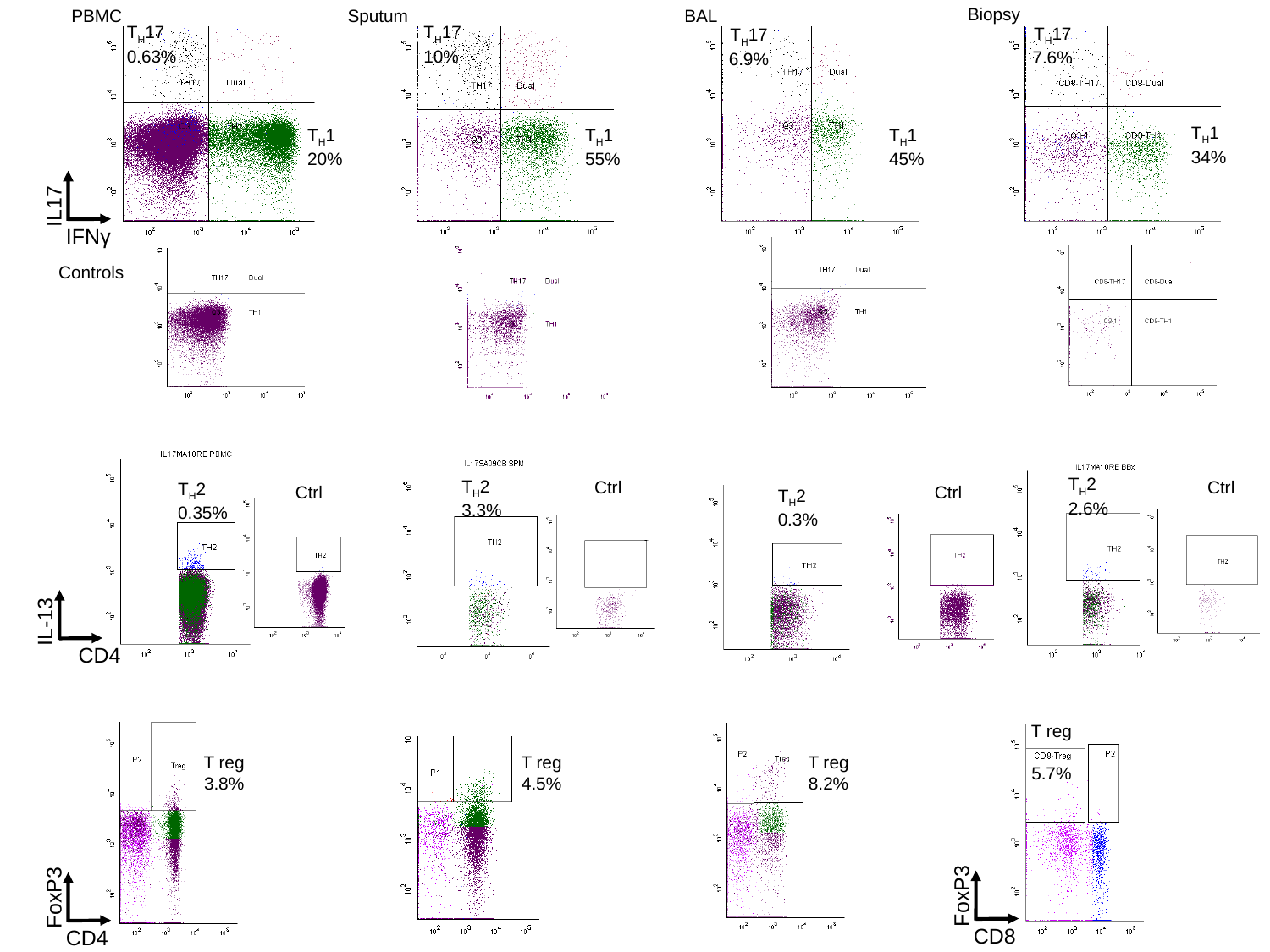

Biopsy
PBMC
Sputum
BAL
TH17
0.63%
TH17
10%
TH17
7.6%
TH17
6.9%
TH1
34%
TH1
20%
TH1
55%
TH1
45%
IL17
IFNγ
Controls
TH2
2.6%
TH2
3.3%
Ctrl
Ctrl
TH2
0.35%
Ctrl
Ctrl
TH2
0.3%
IL-13
CD4
T reg
5.7%
T reg
3.8%
T reg
4.5%
T reg
8.2%
FoxP3
FoxP3
CD8
CD4
