# Supplementary material for: Innate and adaptive T cells in asthmatic patients: Relationship to severity and disease mechanisms
Source: J Allergy Clin Immunol. 2015 Aug;136(2):323–33. doi: 10.1016/j.jaci.2015.01.014 (PMC4534770; doi:10.1016/j.jaci.2015.01.014)
Supplement: Fig E3 [file mmc4.ppt]

## Slide 1
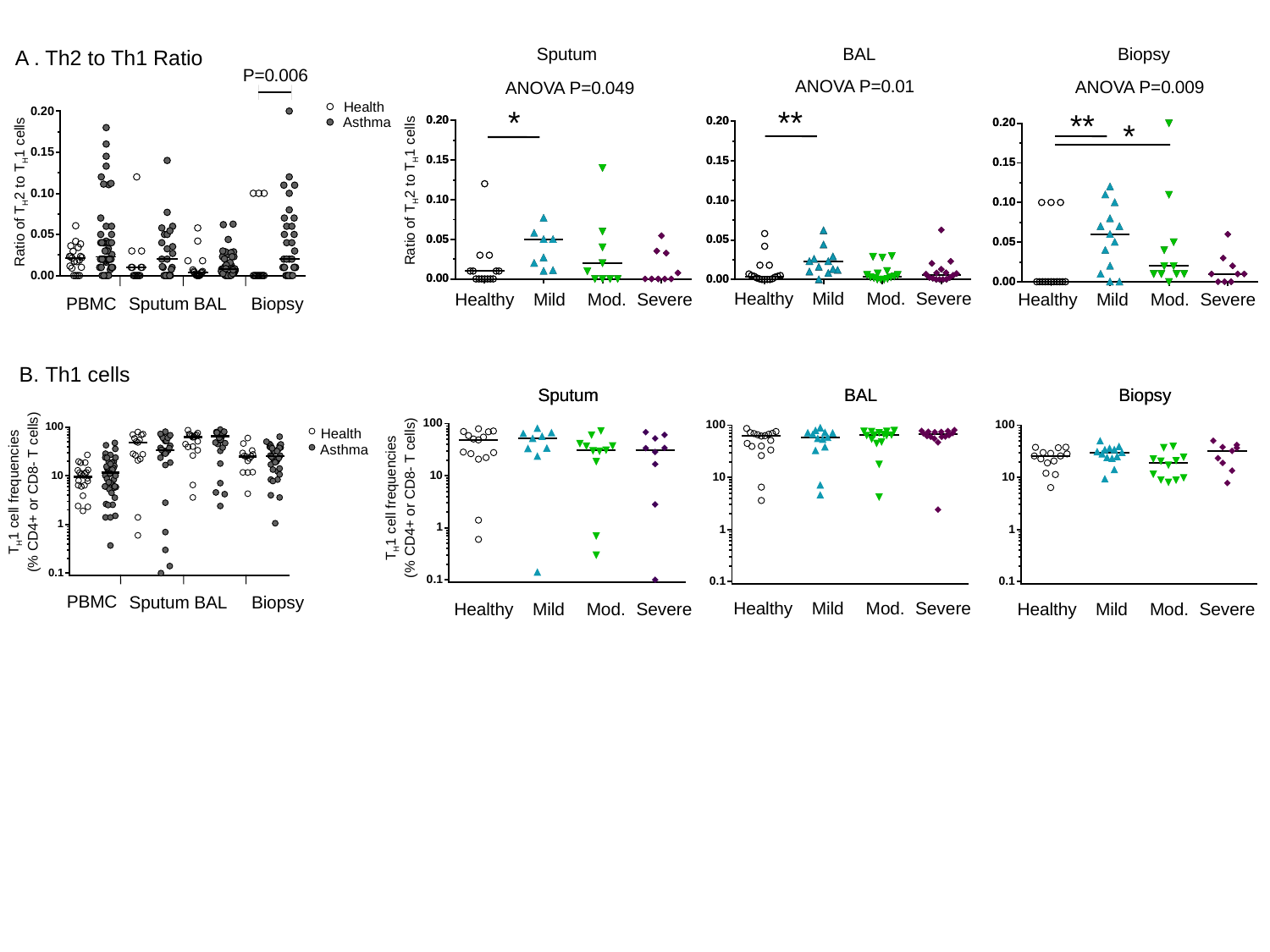

Sputum
BAL
Biopsy
A . Th2 to Th1 Ratio
P=0.006
ANOVA P=0.01
ANOVA P=0.009
ANOVA P=0.049
Health
*
**
**
Asthma
*
Ratio of TH2 to TH1 cells
Ratio of TH2 to TH1 cells
Healthy
Mild
Mod.
Severe
Healthy
Healthy
Mild
Mod.
Severe
Mild
Mod.
Severe
PBMC
Sputum
BAL
Biopsy
B. Th1 cells
Sputum
Sputum
BAL
BAL
Biopsy
Biopsy
Health
Asthma
TH1 cell frequencies
(% CD4+ or CD8- T cells)
TH1 cell frequencies
(% CD4+ or CD8- T cells)
PBMC
Sputum
BAL
Biopsy
Healthy
Mild
Mod.
Severe
Healthy
Mild
Mod.
Severe
Healthy
Mild
Mod.
Severe
