# Supplementary material for: Innate and adaptive T cells in asthmatic patients: Relationship to severity and disease mechanisms
Source: J Allergy Clin Immunol. 2015 Aug;136(2):323–33. doi: 10.1016/j.jaci.2015.01.014 (PMC4534770; doi:10.1016/j.jaci.2015.01.014)
Supplement: Fig E4 [file mmc5.ppt]

## Slide 1
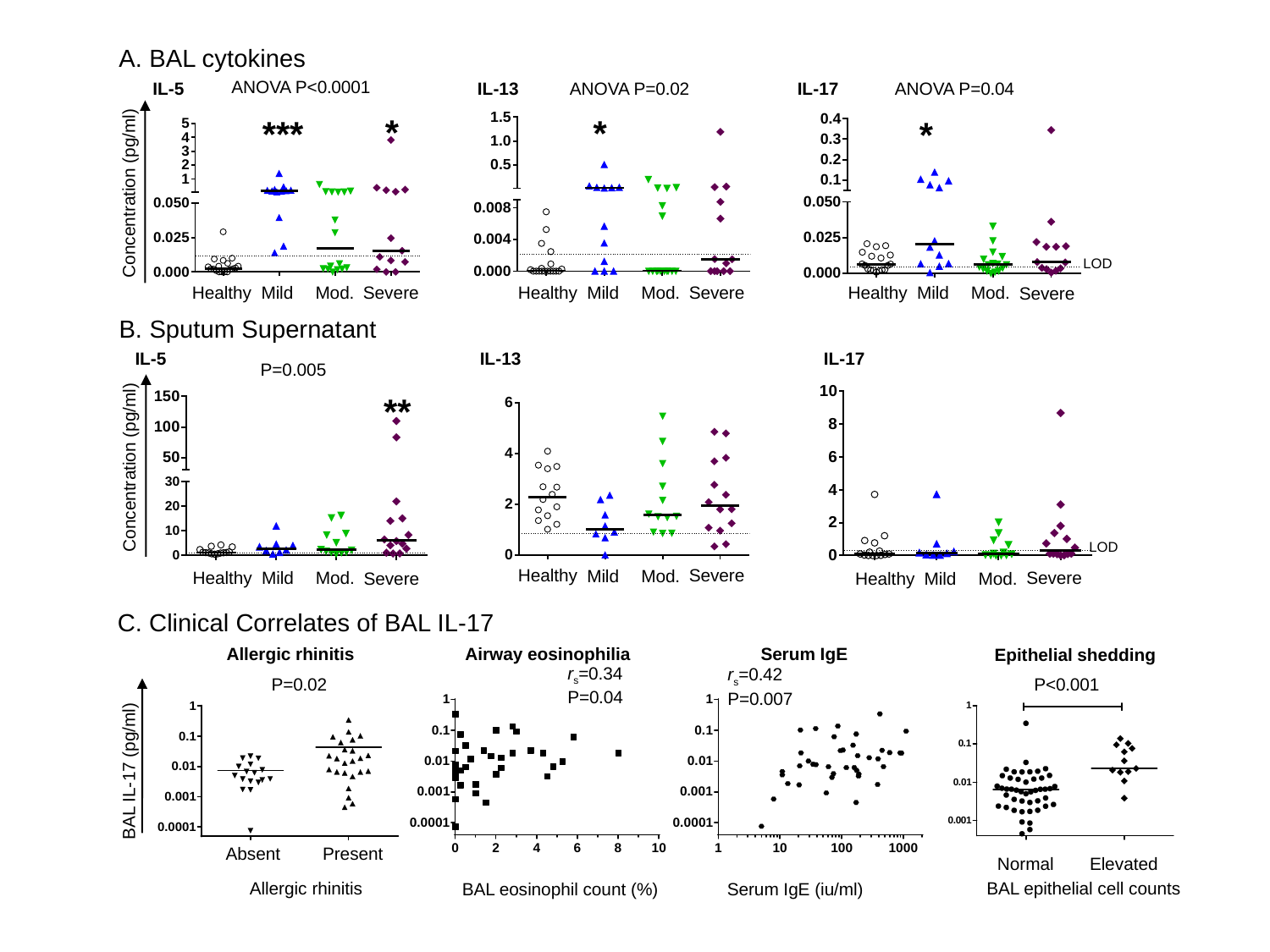

A. BAL cytokines
ANOVA P<0.0001
IL-17
IL-5
IL-13
ANOVA P=0.02
ANOVA P=0.04
*
*
***
*
Concentration (pg/ml)
LOD
Healthy
Mild
Mod.
Healthy
Mild
Mod.
Severe
Severe
Healthy
Mild
Mod.
Severe
B. Sputum Supernatant
IL-5
IL-13
IL-17
P=0.005
**
Concentration (pg/ml)
LOD
Healthy
Severe
Mild
Mod.
Severe
Healthy
Mild
Mod.
Severe
Healthy
Mild
Mod.
C. Clinical Correlates of BAL IL-17
Serum IgE
Allergic rhinitis
Airway eosinophilia
Epithelial shedding
rs=0.34
P=0.04
rs=0.42
P=0.007
P=0.02
P<0.001
BAL IL-17 (pg/ml)
Absent
Present
Normal
Elevated
Allergic rhinitis
BAL epithelial cell counts
BAL eosinophil count (%)
Serum IgE (iu/ml)
