# Supplementary material for: Innate and adaptive T cells in asthmatic patients: Relationship to severity and disease mechanisms
Source: J Allergy Clin Immunol. 2015 Aug;136(2):323–33. doi: 10.1016/j.jaci.2015.01.014 (PMC4534770; doi:10.1016/j.jaci.2015.01.014)
Supplement: Fig E5 [file mmc6.ppt]

## Slide 1
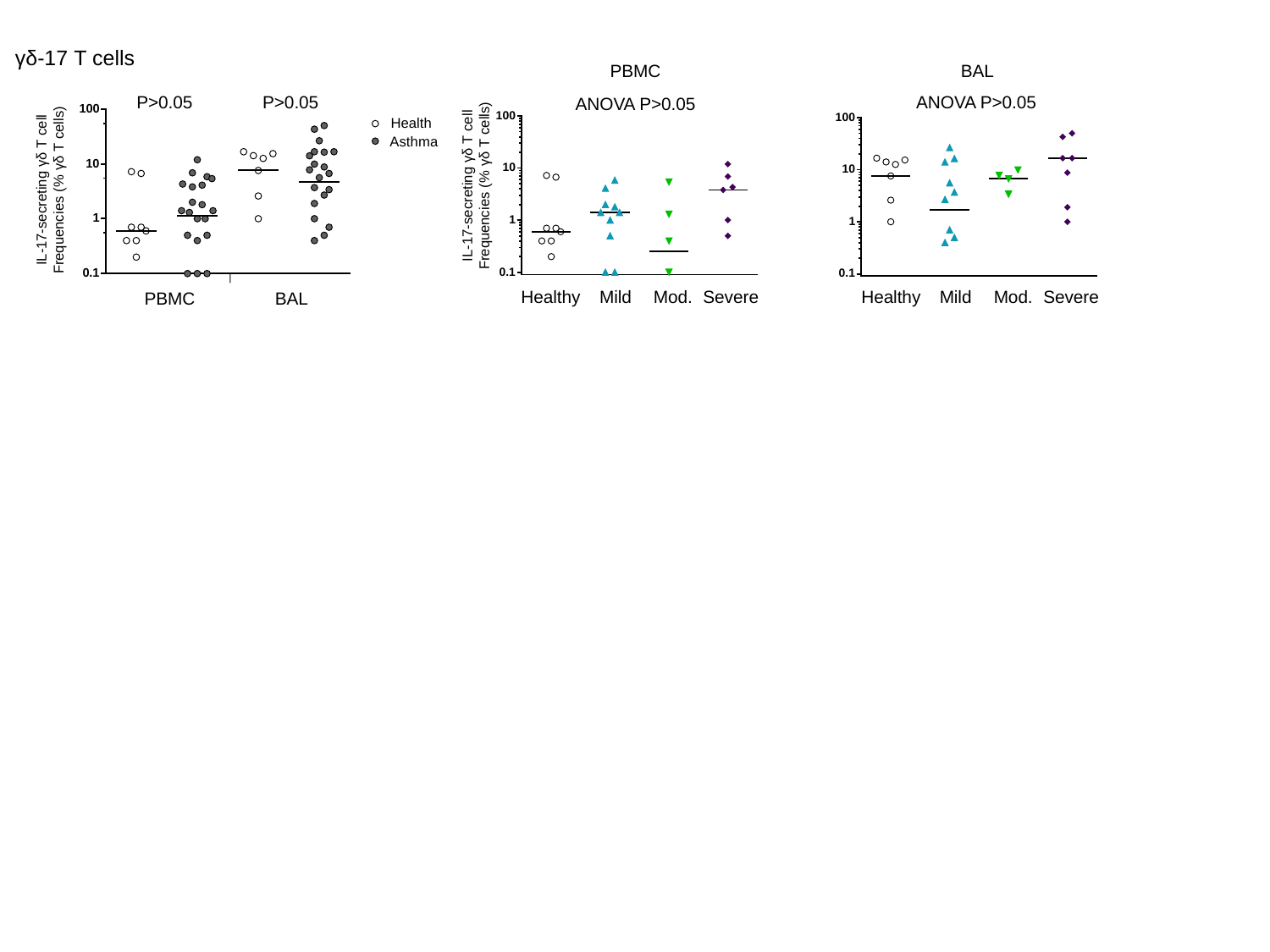

γδ-17 T cells
PBMC
BAL
P>0.05
P>0.05
ANOVA P>0.05
ANOVA P>0.05
Health
Asthma
IL-17-secreting γδ T cell
Frequencies (% γδ T cells)
IL-17-secreting γδ T cell
Frequencies (% γδ T cells)
Healthy
Mild
Mod.
Severe
Healthy
Mild
Mod.
Severe
PBMC
BAL
