# Supplementary material for: Innate and adaptive T cells in asthmatic patients: Relationship to severity and disease mechanisms
Source: J Allergy Clin Immunol. 2015 Aug;136(2):323–33. doi: 10.1016/j.jaci.2015.01.014 (PMC4534770; doi:10.1016/j.jaci.2015.01.014)
Supplement: Fig E6 [file mmc7.ppt]

## Slide 1
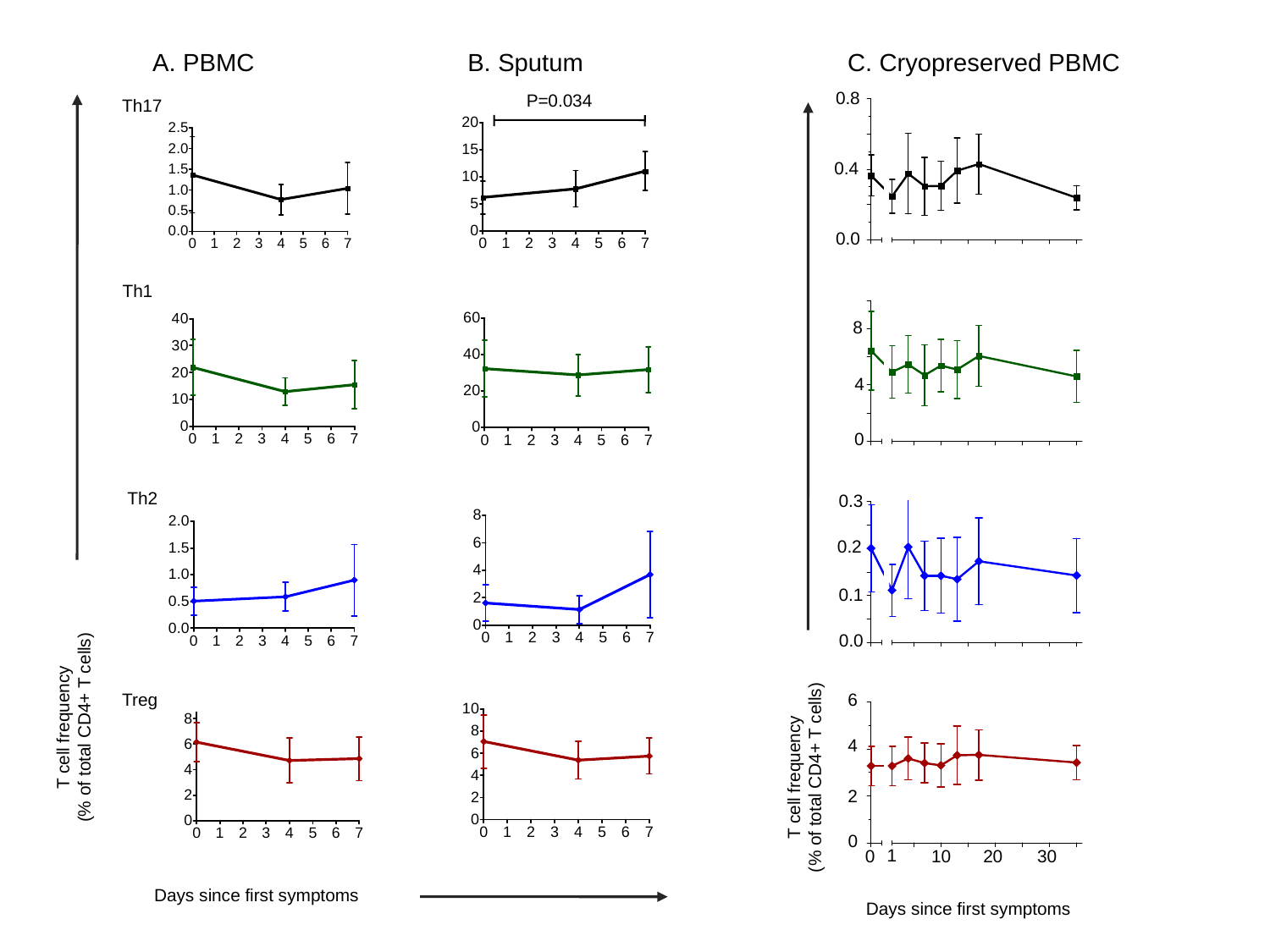

A. PBMC
B. Sputum
C. Cryopreserved PBMC
0.8
P=0.034
Th17
0.4
0.0
Th1
8
4
0
Th2
0.3
0.2
0.1
0.0
Treg
6
T cell frequency
(% of total CD4+ T cells)
4
T cell frequency
(% of total CD4+ T cells)
2
0
1
0
10
20
30
Days since first symptoms
Days since first symptoms
