# Supplementary material for: Innate and adaptive T cells in asthmatic patients: Relationship to severity and disease mechanisms
Source: J Allergy Clin Immunol. 2015 Aug;136(2):323–33. doi: 10.1016/j.jaci.2015.01.014 (PMC4534770; doi:10.1016/j.jaci.2015.01.014)
Supplement: Fig E7 [file mmc8.ppt]

## Slide 1
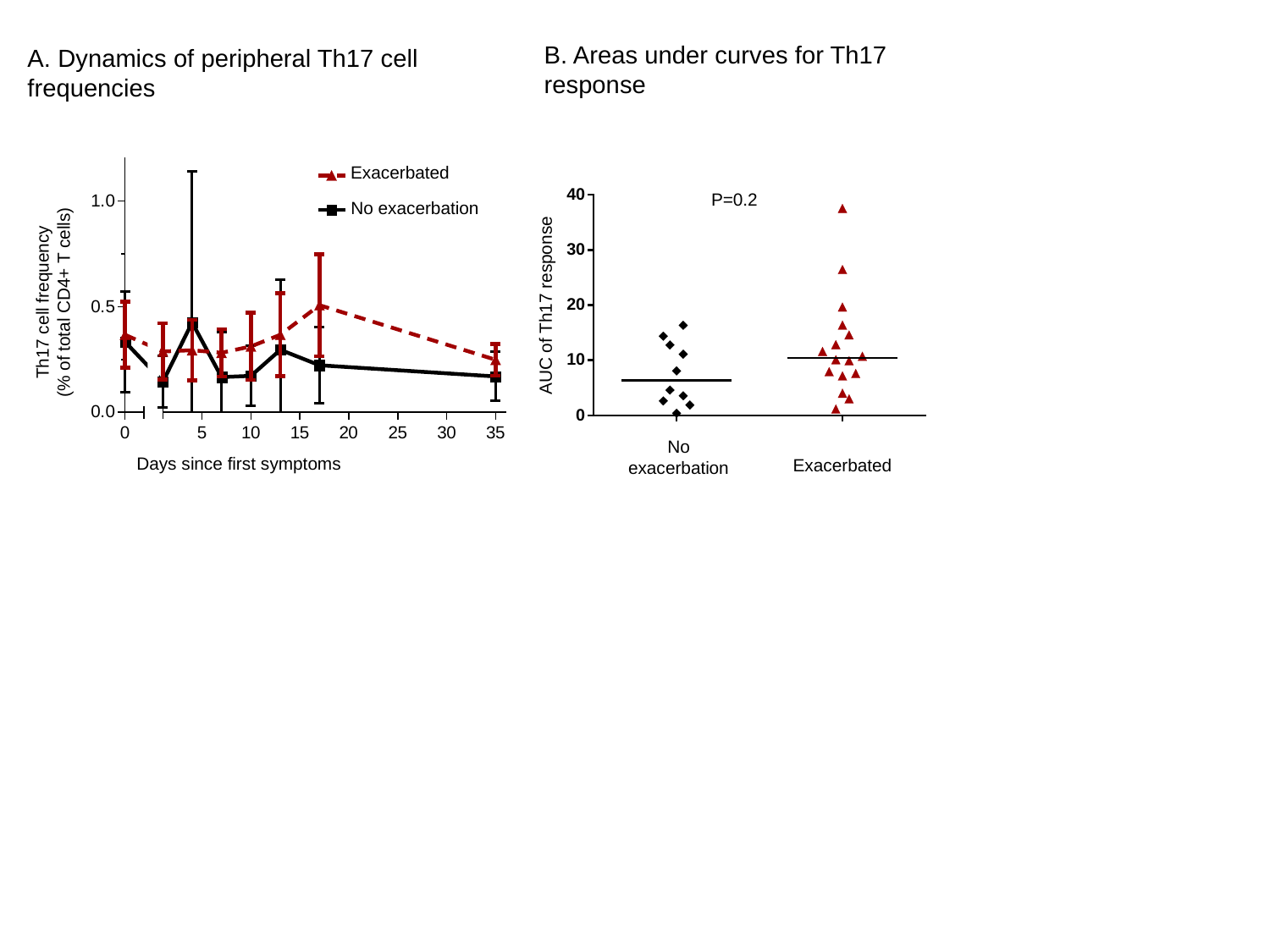

B. Areas under curves for Th17 response
A. Dynamics of peripheral Th17 cell frequencies
Exacerbated
P=0.2
No exacerbation
Th17 cell frequency
(% of total CD4+ T cells)
AUC of Th17 response
No
exacerbation
Days since first symptoms
Exacerbated
