# Supplementary material for: Innate and adaptive T cells in asthmatic patients: Relationship to severity and disease mechanisms
Source: J Allergy Clin Immunol. 2015 Aug;136(2):323–33. doi: 10.1016/j.jaci.2015.01.014 (PMC4534770; doi:10.1016/j.jaci.2015.01.014)
Supplement: Fig E8 [file mmc9.ppt]

## Slide 1
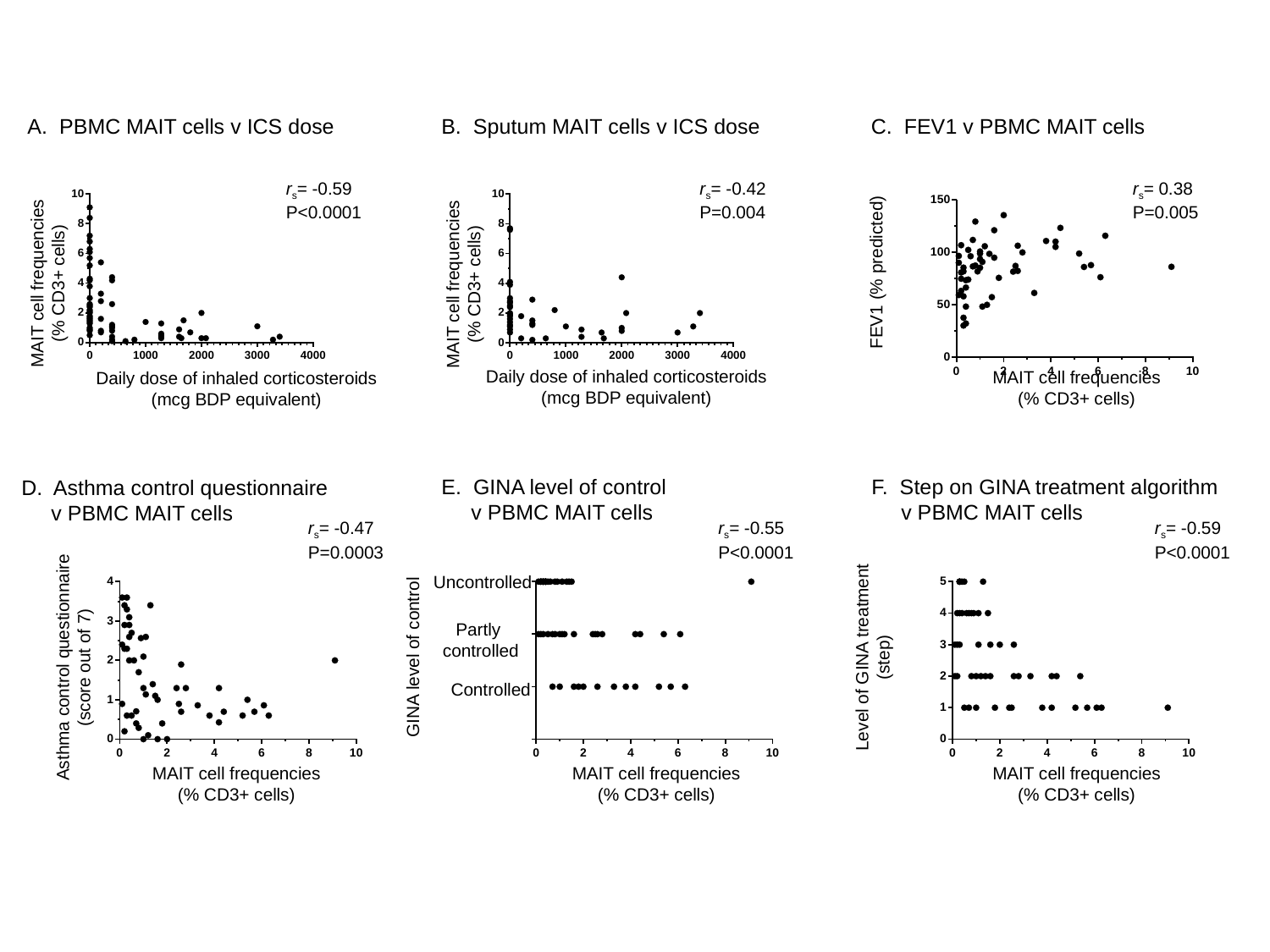

B. Sputum MAIT cells v ICS dose
C. FEV1 v PBMC MAIT cells
A. PBMC MAIT cells v ICS dose
rs= -0.59
P<0.0001
rs= -0.42
P=0.004
rs= 0.38
P=0.005
FEV1 (% predicted)
MAIT cell frequencies
(% CD3+ cells)
MAIT cell frequencies
(% CD3+ cells)
Daily dose of inhaled corticosteroids
(mcg BDP equivalent)
MAIT cell frequencies
(% CD3+ cells)
Daily dose of inhaled corticosteroids
(mcg BDP equivalent)
E. GINA level of control
 v PBMC MAIT cells
F. Step on GINA treatment algorithm
 v PBMC MAIT cells
Asthma control questionnaire
 v PBMC MAIT cells
rs= -0.47
P=0.0003
rs= -0.55
P<0.0001
rs= -0.59
P<0.0001
Uncontrolled
Partly
controlled
Level of GINA treatment
(step)
Asthma control questionnaire
(score out of 7)
GINA level of control
Controlled
MAIT cell frequencies
(% CD3+ cells)
MAIT cell frequencies
(% CD3+ cells)
MAIT cell frequencies
(% CD3+ cells)
