# Supplementary material for: Innate and adaptive T cells in asthmatic patients: Relationship to severity and disease mechanisms
Source: J Allergy Clin Immunol. 2015 Aug;136(2):323–33. doi: 10.1016/j.jaci.2015.01.014 (PMC4534770; doi:10.1016/j.jaci.2015.01.014)
Supplement: Fig E9 [file mmc10.ppt]

## Slide 1
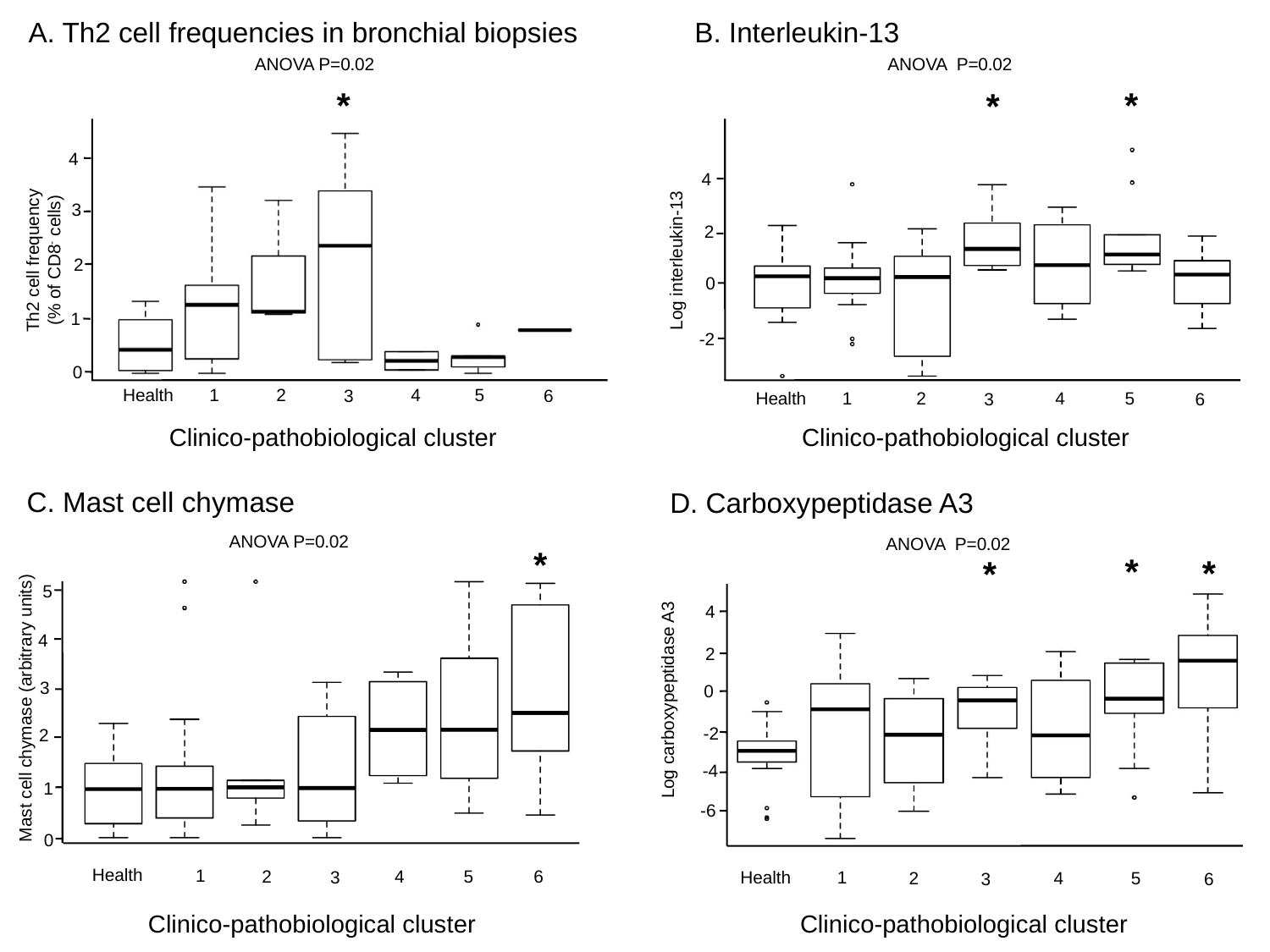

A. Th2 cell frequencies in bronchial biopsies
B. Interleukin-13
ANOVA P=0.02
ANOVA P=0.02
*
*
*
4
4
3
2
Th2 cell frequency
(% of CD8- cells)
Log interleukin-13
2
0
1
-2
0
1
Health
5
2
4
6
3
1
Health
5
2
4
6
3
Clinico-pathobiological cluster
Clinico-pathobiological cluster
C. Mast cell chymase
D. Carboxypeptidase A3
ANOVA P=0.02
ANOVA P=0.02
*
*
*
*
5
4
4
2
3
0
Log carboxypeptidase A3
Mast cell chymase (arbitrary units)
-2
2
-4
1
-6
0
Health
1
5
2
4
6
3
1
Health
5
2
4
6
3
Clinico-pathobiological cluster
Clinico-pathobiological cluster
