# Supplementary material for: Innate and adaptive T cells in asthmatic patients: Relationship to severity and disease mechanisms
Source: J Allergy Clin Immunol. 2015 Aug;136(2):323–33. doi: 10.1016/j.jaci.2015.01.014 (PMC4534770; doi:10.1016/j.jaci.2015.01.014)
Supplement: Fig E10 [file mmc11.ppt]

## Slide 1
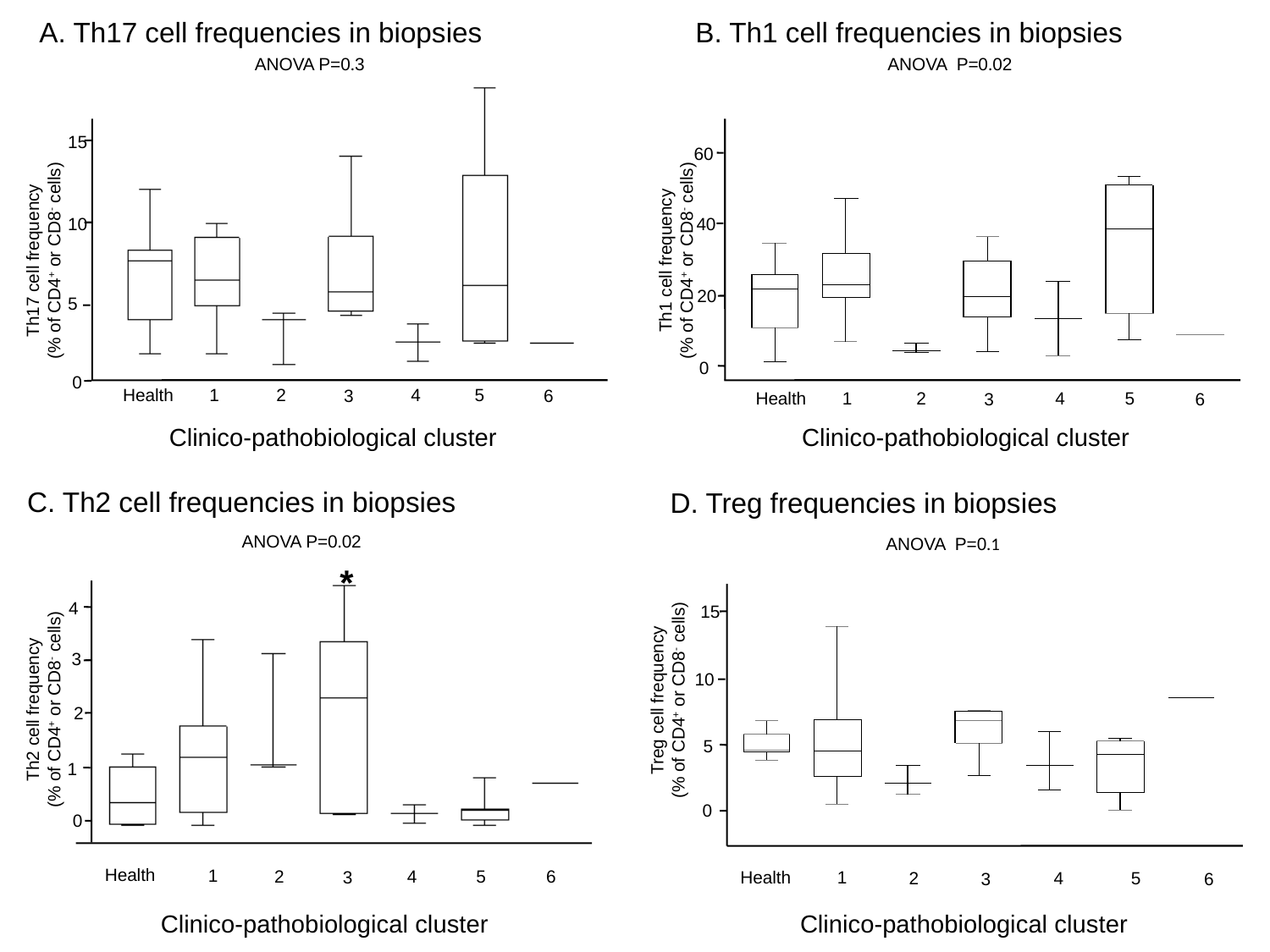

A. Th17 cell frequencies in biopsies
B. Th1 cell frequencies in biopsies
ANOVA P=0.3
ANOVA P=0.02
15
60
10
40
Th17 cell frequency
(% of CD4+ or CD8- cells)
Th1 cell frequency
(% of CD4+ or CD8- cells)
20
5
0
0
1
Health
5
2
4
6
3
1
Health
5
2
4
6
3
Clinico-pathobiological cluster
Clinico-pathobiological cluster
C. Th2 cell frequencies in biopsies
D. Treg frequencies in biopsies
ANOVA P=0.02
ANOVA P=0.1
*
4
15
3
10
Treg cell frequency
(% of CD4+ or CD8- cells)
Th2 cell frequency
(% of CD4+ or CD8- cells)
2
5
1
0
0
Health
1
5
2
4
6
3
1
Health
5
2
4
6
3
Clinico-pathobiological cluster
Clinico-pathobiological cluster
