# Supplementary material for: Innate and adaptive T cells in asthmatic patients: Relationship to severity and disease mechanisms
Source: J Allergy Clin Immunol. 2015 Aug;136(2):323–33. doi: 10.1016/j.jaci.2015.01.014 (PMC4534770; doi:10.1016/j.jaci.2015.01.014)
Supplement: Fig E11 [file mmc12.ppt]

## Slide 1
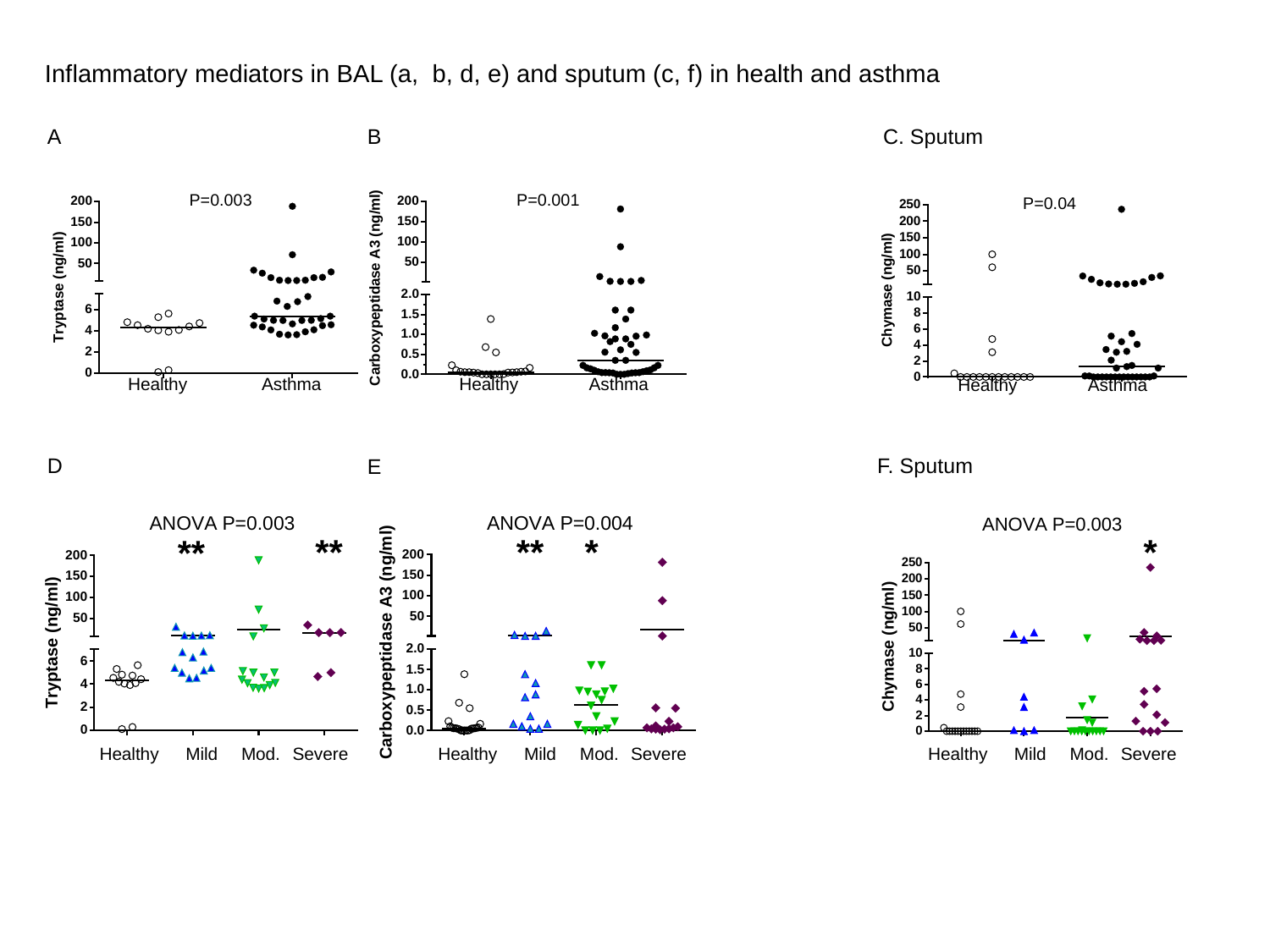

Inflammatory mediators in BAL (a, b, d, e) and sputum (c, f) in health and asthma
A
B
C. Sputum
Healthy
Healthy
Asthma
Asthma
Healthy
Asthma
D
F. Sputum
E
**
**
*
*
**
Healthy
Healthy
Healthy
Mild
Mod.
Severe
Mild
Mod.
Severe
Mild
Mod.
Severe
