# Supplementary material for: Innate and adaptive T cells in asthmatic patients: Relationship to severity and disease mechanisms
Source: J Allergy Clin Immunol. 2015 Aug;136(2):323–33. doi: 10.1016/j.jaci.2015.01.014 (PMC4534770; doi:10.1016/j.jaci.2015.01.014)
Supplement: Fig E12 [file mmc13.ppt]

## Slide 1
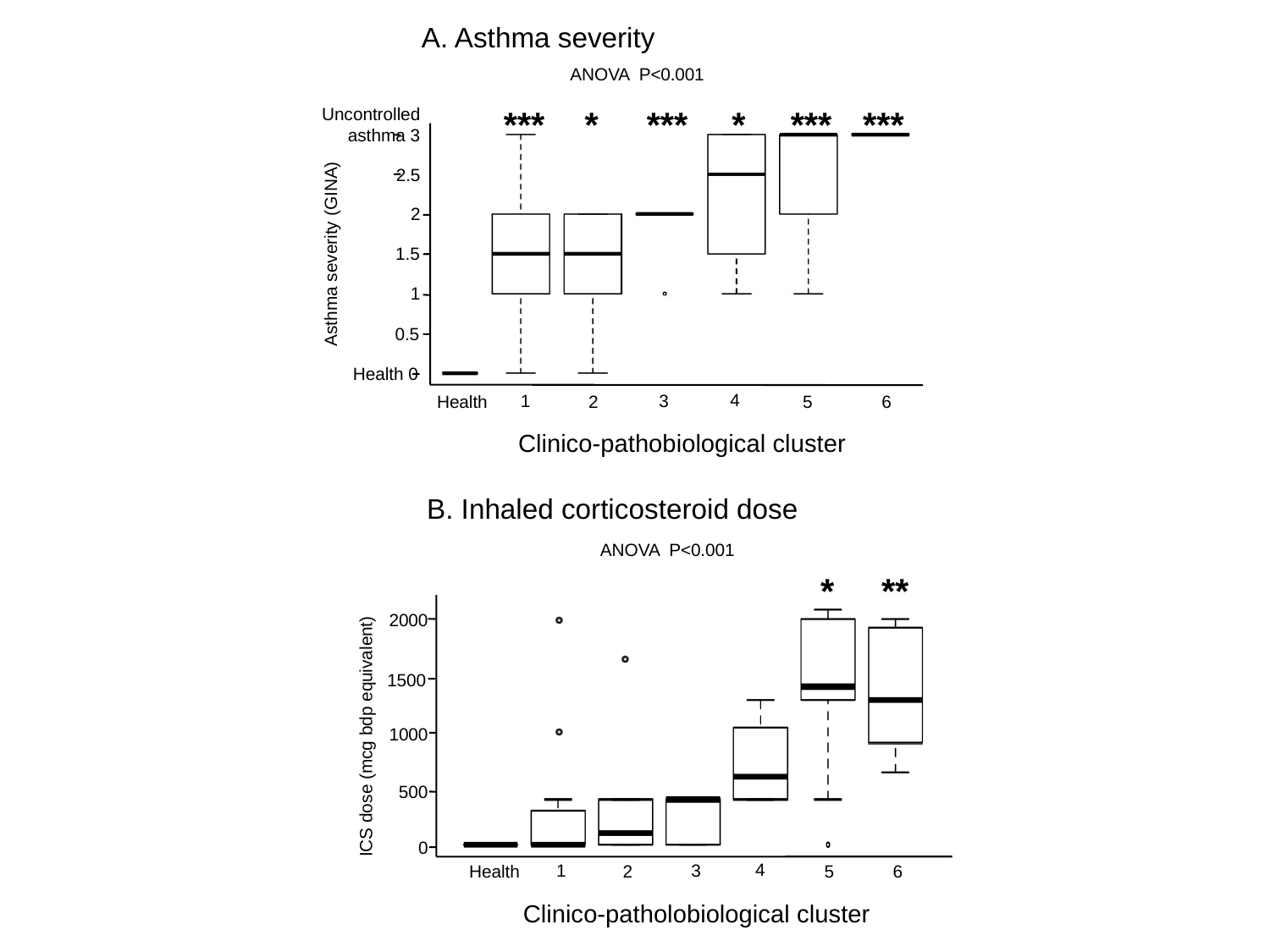

A. Asthma severity
ANOVA P<0.001
Uncontrolled asthma 3
***
*
***
*
***
***
2.5
2
1.5
Asthma severity (GINA)
1
0.5
Health 0
4
3
1
Health
5
2
6
Clinico-pathobiological cluster
B. Inhaled corticosteroid dose
ANOVA P<0.001
*
**
2000
1500
1000
ICS dose (mcg bdp equivalent)
500
0
4
3
1
Health
5
2
6
Clinico-patholobiological cluster
