# Supplementary material for: Innate and adaptive T cells in asthmatic patients: Relationship to severity and disease mechanisms
Source: J Allergy Clin Immunol. 2015 Aug;136(2):323–33. doi: 10.1016/j.jaci.2015.01.014 (PMC4534770; doi:10.1016/j.jaci.2015.01.014)
Supplement: Fig E13 [file mmc14.ppt]

## Slide 1
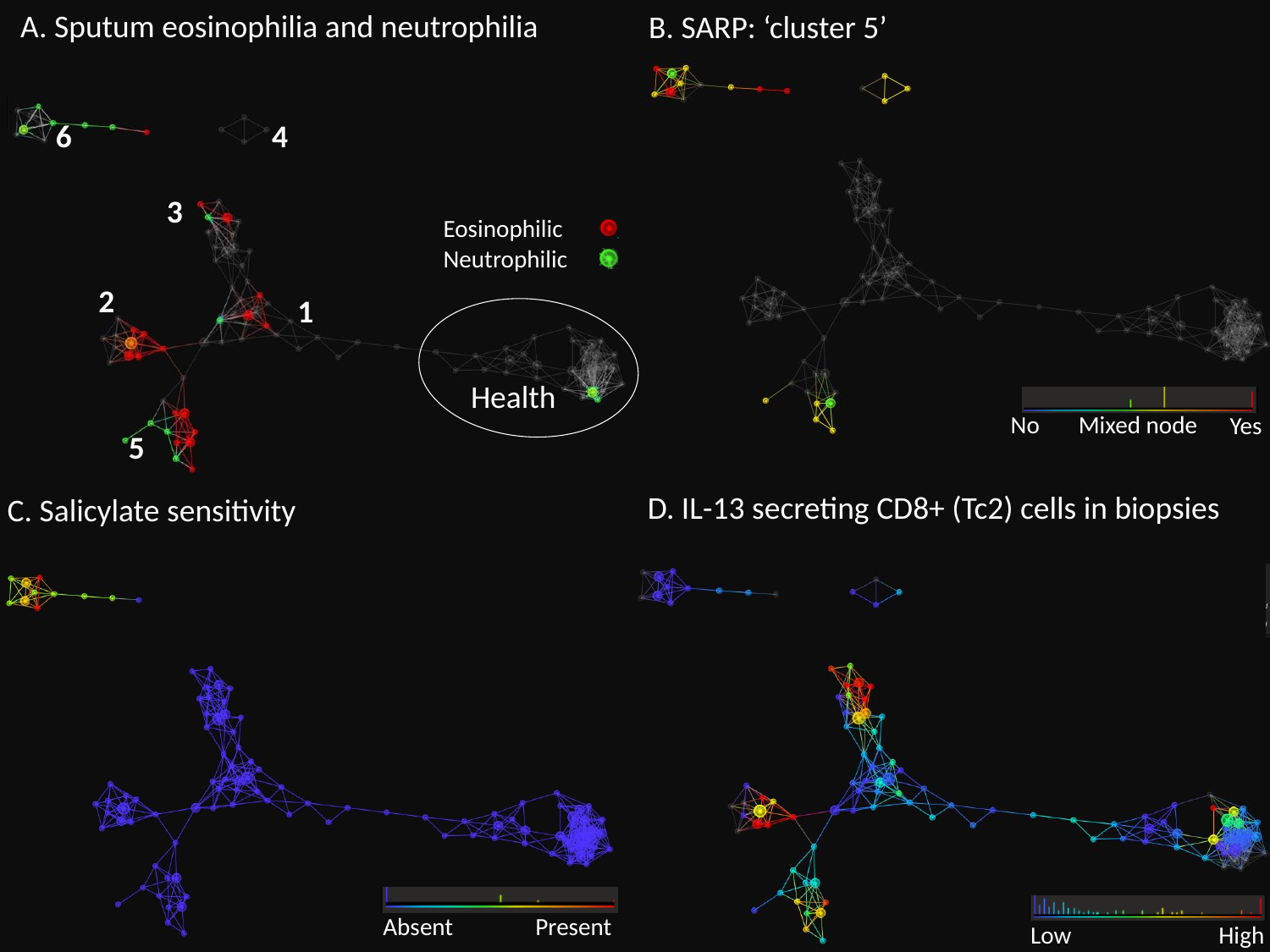

A. Sputum eosinophilia and neutrophilia
B. SARP: ‘cluster 5’
6
4
3
Eosinophilic
Neutrophilic
2
1
 Health
No
Mixed node
Yes
5
D. IL-13 secreting CD8+ (Tc2) cells in biopsies
C. Salicylate sensitivity
Absent
Present
Low
High
