# Supplementary material for: Innate and adaptive T cells in asthmatic patients: Relationship to severity and disease mechanisms
Source: J Allergy Clin Immunol. 2015 Aug;136(2):323–33. doi: 10.1016/j.jaci.2015.01.014 (PMC4534770; doi:10.1016/j.jaci.2015.01.014)
Supplement: Fig E14 [file mmc15.ppt]

## Slide 1
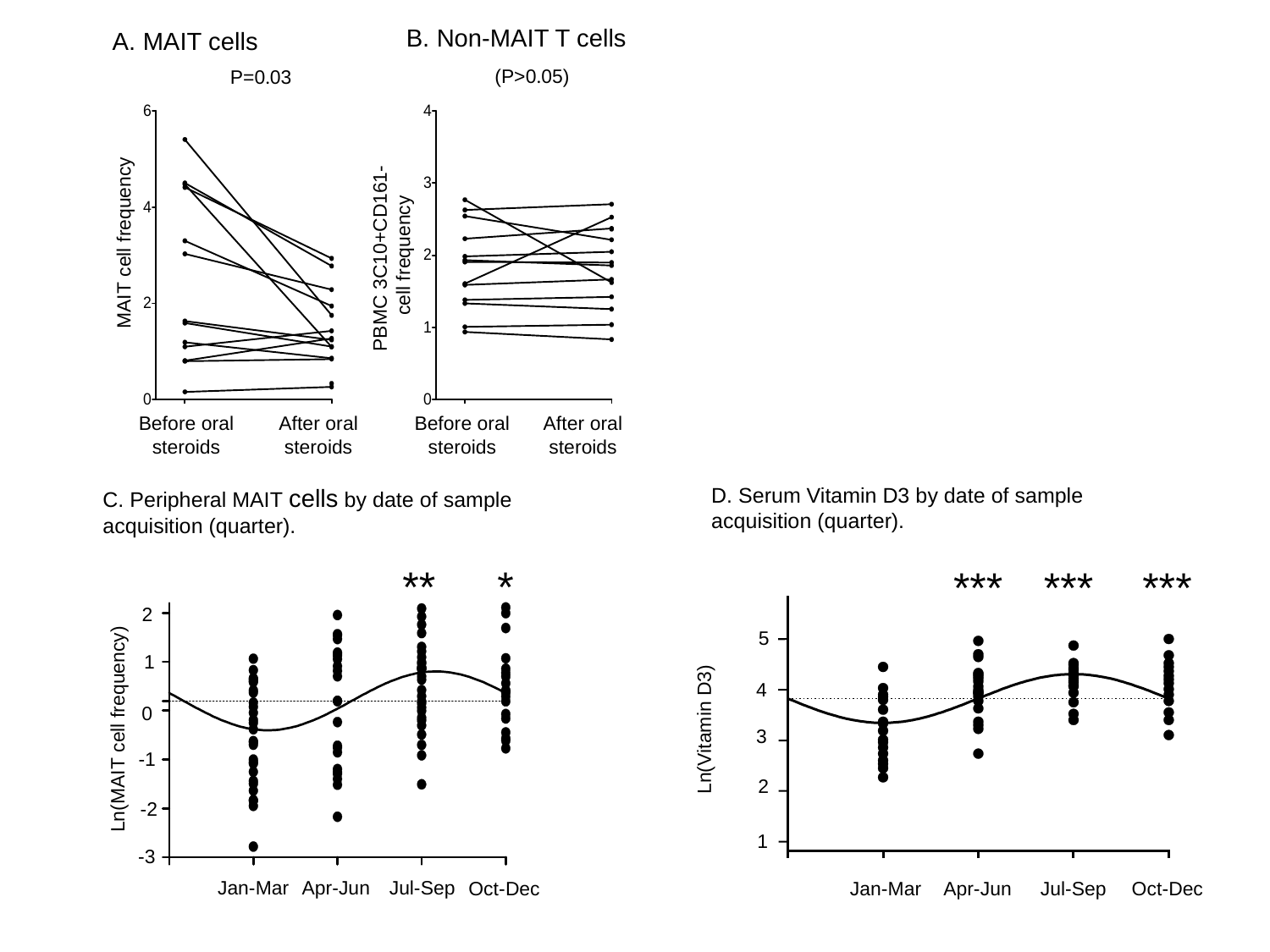

B. Non-MAIT T cells
A. MAIT cells
(P>0.05)
P=0.03
MAIT cell frequency
PBMC 3C10+CD161-
cell frequency
Before oral
steroids
After oral
steroids
Before oral
steroids
After oral
steroids
C. Peripheral MAIT cells by date of sample acquisition (quarter).
D. Serum Vitamin D3 by date of sample acquisition (quarter).
**
*
***
***
***
2
5
1
4
0
Ln(Vitamin D3)
Ln(MAIT cell frequency)
3
-1
2
-2
1
-3
Jan-Mar
Apr-Jun
Jul-Sep
Oct-Dec
Jan-Mar
Apr-Jun
Jul-Sep
Oct-Dec
